# High-affinity SOAT1 ligands remodeled cholesterol metabolism program to inhibit tumor growth

**DOI:** 10.1186/s12916-022-02436-8

**Published:** 2022-08-09

**Authors:** Zhihua Wang, Miaomiao Wang, Mengxin Zhang, Kaikun Xu, Xinshuai Zhang, Yi Xie, Yiming Zhang, Cheng Chang, Xiaolu Li, Aihua Sun, Fuchu He

**Affiliations:** 1 State Key Laboratory of Proteomics, National Center for Protein Sciences (Beijing), Beijing Proteome Research Center, Beijing Institute of Lifeomics, Beijing, 102206 China; 2 Research Unit of Proteomics Dirven Cancer Precision Medicine, Chinese Academy of Medical Sciences, Beijing, 102206 China; 3 Shandong First Medical University, The First Affiliated Hospital of Shandong First Medical University, Jinan, 250014 China; 4 Department of Pharmacology and Pharmaceutical Sciences, School of Medicine, Tsinghua University, Beijing, 100083 China

**Keywords:** Cholesterol metabolism, Hepatocelluar carcinoma, SOAT1, Nilotinib, Antitumor mechanism

## Abstract

**Background:**

Although cholesterol metabolism is a common pathway for the development of antitumor drugs, there are no specific targets and drugs for clinical use. Here, based on our previous study of sterol O-acyltransferase 1 (SOAT1) in hepatocelluar carcinoma, we sought to screen an effective targeted drug for precise treatment of hepatocelluar carcinoma and, from the perspective of cholesterol metabolism, clarify the relationship between cholesterol regulation and tumorigenesis and development.

**Methods:**

In this study, we developed a virtual screening integrated affinity screening technology for target protein drug screening. A series of in vitro and in vivo experiments were used for drug activity verification. Multi-omics analysis and flow cytometry analysis were used to explore antitumor mechanisms. Comparative analysis of proteome and transcriptome combined with survival follow-up information of patients reveals the clinical therapeutic potential of screened drugs.

**Results:**

We screened three compounds, nilotinib, ABT-737, and evacetrapib, that exhibited optimal binding with SOAT1. In particular, nilotinib displayed a high affinity for SOAT1 protein and significantly inhibited tumor activity both in vitro and in vivo. Multi-omics analysis and flow cytometry analysis indicated that SOAT1-targeting compounds reprogrammed the cholesterol metabolism in tumors and enhanced CD8^+^ T cells and neutrophils to suppress tumor growth.

**Conclusions:**

Taken together, we reported several high-affinity SOAT1 ligands and demonstrated their clinical potential in the precision therapy of liver cancer, and also reveal the potential antitumor mechanism of SOAT1-targeting compounds.

**Graphical Abstract:**

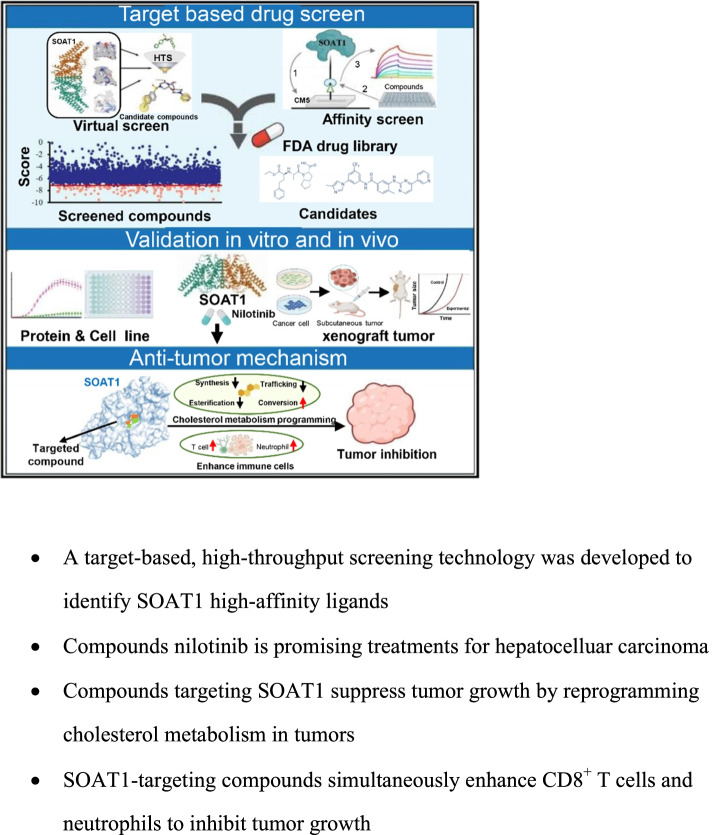

**Supplementary Information:**

The online version contains supplementary material available at 10.1186/s12916-022-02436-8.

## Background

Previous studies have found that reprogramming of tumor cholesterol metabolism occurs during tumorigenesis and development [1, 2]. Characteristics of the cholesterol metabolism of tumors include both the upregulation of the synthesis and uptake of cholesterol and the accumulation of various cholesterol derivatives, such as cholesterol esters and oxidized cholesterol [3]. However, the reprogramming of the cholesterol metabolism in tumors involves many processes, including synthesis, uptake, esterification, efflux, and conversion. Thus, elucidating the role of these processes in tumorigenesis and in the development of potential therapeutic drugs has been challenging.

Many recent clinical and preclinical studies have shown that targeting the cholesterol metabolism in tumor cells and immune cells is an obvious way to treat tumors [4]. Existing targets include key enzymes in the cholesterol synthesis and transport pathways, as well as key transcription factors involved in cholesterol metabolism. For example, statins exert anticancer effects by inhibiting the activity of the key rate-limiting enzyme 3-hydroxy-3-methylglutaryl-coenzyme A reductase (HMGCR) in the cholesterol synthesis signaling pathway [5], and itraconazole directly binds to the sterol-sensitive domain of NPC intracellular cholesterol transporter 1 (NPC1), thereby reducing tumor growth and angiogenesis [6]. In addition, LXR-623, GW3965, other small-molecule inhibitors against liver X receptor (LXR), and other small-molecule drugs are also currently being investigated as antitumor drugs [7].

The discovery of new cholesterol-related drug targets is expected to accelerate the development of drugs targeting intracellular cholesterol homeostasis for tumor therapy. SOAT1 protein can convert excess intracellular cholesterol into cholesterol ester and store in lipid droplets. Knockdown or inhibition of SOAT1 can inhibit a variety of tumors by regulating cholesterol homeostasis in tumor cells [8, 9], and the immune state of the tumor microenvironment [10, 11]. We previously found that SOAT1 can be used to stratify HCC patients with poor prognosis, and as a drug target for the treatment of patients with this subtype. Inhibitors targeting SOAT1 have been shown to exert optimal antitumor effects both in vitro and in vivo. However, there are only a few known SOAT1 inhibitors—only avasimibe, nevanimibe, and CI-976 have been reported—which greatly limits its drug development prospects.

Recognizing the potential of SOAT1 protein in tumor therapy, several international teams have successively reported the precise crystal structure of SOAT1, which provides a new opportunity for target protein-based drug development [12, 13]. The primary aim of this research was to continue the preliminary work of the laboratory and develop a high-throughput, ligand screening technology for the identification of potential therapeutic targets of SOAT1 protein for S-III liver cancer patients. Furthermore, we investigated the intrinsic link between cholesterol regulation and tumorigenesis and development in order to provide new insights for the precise treatment of liver cancer.

## Methods

### Cell lines and cell culture conditions

The human hepatoellular carcinomas cell line HepG2, Hep3B, and Huh7 were obtained from ACTT with ACTTnumber: HB-8065, HB-8064, and PTA-4583. The mouse hepatoellular carcinoma cell line Hepa1-6 was obtained from ACTT with ACTTnumber: CRL-1830. The HEK293T cells were obtained from ACTT with ACTTnumber: CRL-3216. The Expi293F™ cells were obtained from Thermo Scientific with Cat#A14527. All of the cells were cultured in Dulbecco’s modified Eagle’s media (DMEM; Thermo Fisher Scientific) containing 10% fetal bovine serum (FBS; Thermo Fisher Scientific), 100 μg/mL streptomycin, and 100 units/mL penicillin (Thermo Fisher Scientific) at 37 °C with 5% CO_2_. Cells were passaged or harvested by incubation with 1 × TrypLE Express (Life Technologies) at 37 °C for 2 and 5 min.

### Animal studies

The animal care and experimental protocols were approved by the Institutional Animal Care and Use Committee (IACUC) of National Center for Protein Sciences (Beijing), Ethical review number: IACUC-20210914-32MT. NOD-SCID and C57BL/6 J mice were purchased from Charles River, Inc (Beijing, Vital River Laboratory Animal Technology). All mice were female mice of 4–5 weeks. After tumor transplantation, the tumor grew to 100 mm^3^ and started to randomly divided into three groups: control (200 μL-30% PEG400 + 0.5% Tween 80 + 5% propylene glycol, intraperitoneal injection), sorafenib (20 mg kg^−1^ day^−1^, intraperitoneal injection), nilotinib (20 mg kg^−1^ day^−1^, intraperitoneal injection). After treatment for 28 days, the mice were killed by spinal dislocation. The investigator was blinded to group medication during the experiment and when assessing the outcome. Animals were housed in a specific pathogen-free mouse facility at the animal center National Center for Protein Sciences (Beijing). Maximal tumor size of CDX mouse model of experimental endpoint is less than 20 mm in any dimension, in accordance with the IACUC. None of the guidelines was exceeded in any experiment performed. Tumor size was measured using a caliper. Tumor volume in mm^3^ was calculated using the formula: tumor volume = 0.5 × length × (width)^2^.

### Targeting SOAT1-based virtual screening approaches

The crystal structure of SOAT1 protein was obtained from the Research Collaboration for Structural Bioinformatics Protein Data Bank (RCSB PDB) (protein PDB code: 6VUM and 6L47) [12, 13]. The screening compound database contains 31,276 compounds recognized by the FDA obtained from Selleck (https://www.selleck.cn/screening-libraries). The virtual screening was performed using the previously reported method. Specifically, we used three widely accepted docking software, AutoDock 4.2 [14], sybyl 2.0 [15], and Glide [16] for high-throughput molecular docking. For molecular docking using autoDock, we first charged the protein 3D crystal structure, added polar hydrogens and dehydrated, and energy-optimized, and then created a lattice pocket to accommodate the entire SOAT1 catalytic site. The compounds are also subjected to polar hydrogenation and charge addition, and energy optimization to generate the molecule to be docked. The docking method adopts the flexible docking mode. Then the small molecules in the compound library were docked within the catalytic site pockets through a genetic algorithm and sorted according to the classical free energy function. These input files, including protein structure, active pocket box, and Gasteiger partial charge, were set to default parameters. Sybyl 2.0 docking was performed utilizing the Surflex-dock module. First, the protein and compound library molecules were polarized, hydrogenated, charged, and energy-optimized. The interface bag was centered on the 15-Å rectangle with the coordinates of the His460 residue at the SOAT1 catalytic site. The semi-flexible docking method used the integrated score as the scoring function. The Glide docking module was implemented in Schrödinger version 2015–4. We first used the protein preparation wizard module to add a polar hydrogen, charge, and remove water from the SOAT1 protein. Then, the binding site was defined as a 15-Å rectangle centered on the coordinates of the His460 residue of the SOAT1 catalytic site. At the same time, the LigPrep module with default parameters was utilized to process all screened compounds. For the Glide docking step, we selected standard precision (SP) and super precision (XP) for molecular docking. The ligand docking adopts flexible docking, and the energy is minimized. Finally, we utilized GlideScore as the scoring function. The Glide XP docking posture was utilized to display the final docking result, and the result images were completed by PyMOL 2.3 [17].

### Expression and purification of SOAT1 protein

The SOAT1 protein expression and purification refer to the previously reported method [12, 13]. Specifically, we cloned the cDNA of human SOAT1 (NCBI reference sequence NM_003101.6) into a pCAG vector with a 6 × His-tag at the carboxyl end. The HEK293F suspension cells were cultured in Freestyle 293 medium (Thermo Fisher Scientific) in a constant temperature incubator at 37 °C and provided with 5% CO_2_ and 80% humidity. When the cell density reached 2 × 10^6^ cells per milliliter, the cells were transiently transfected with the expression plasmid and polyethyleneimine. Approximately 1 mg of expression plasmid and 3 mg of polyethyleneimine (Sigma-Aldrich) are pre-mixed in 50 ml of fresh medium and incubated for 15–30 min before transfection. Then 50 ml of the mixture was add to 1 l of cell culture and incubate for 15–30 min. The transfected cells were collected after incubating for 48 h. To purify SOAT1, the collected HEK293F cells were resuspended in a buffer containing 25 mM Tris pH 8.0, 150 mM NaCl (Buffer A), and a protease inhibitor mixture (approximately 1 g of cells plus 5–10 mL of Buffer A). After sonication on ice, the cell membrane pellet was collected and weighed by centrifugation at 120,000* g* in an ultra-high-speed centrifuge at 4 °C for 2 h. The membrane fraction was subjected to 25 mM Tris pH 8.0, 150 mM NaCl, and 1% (w/ v) glyco-diosgenin (GDN, Anatrace) (Buffer B) dissolved for 2 h. After centrifugation at 120,000* g* for 1 h, the supernatant was sought and applied to the nickel column for affinity purification and washed with a washing buffer containing 25 mM Tris pH 8.0, 150 mM NaCl, 40 mM imidazole, and 0.02% GDN (Buffer C). The protein was eluted with a buffer containing 25 mM Tris pH 8.0, 150 mM NaCl, 400 mM imidazole, and 0.02% GDN (Buffer D). Then the eluate was concentrated and purified by size exclusion chromatography (SuperdexTM200 10/300 gL, GE Healthcare) in a buffer containing 25 mM Tris pH 8.0, 150 mM NaCl, and 0.02% GDN (Buffer E), and then purified. The protein concentration and SDS-PAGE detection, the protein concentration, and purity detection through the standard (purity > 95%) can be divided and stored at − 80 ℃ for future use.

### Surface plasmon resonance for affinity screening and affinity determination

A Biacore T200 (GE Healthcare) optical biosensor was used for affinity screening of target proteins and the determination of equilibrium dissociation constant (KD) value measurements for protein–ligand interactions. The CM5 chip (GE Healthcare) was utilized to couple the target protein, and the running buffer was phosphate buffer saline (PBS, Sigma-Aldrich) containing 5% dimethyl sulfoxide (DMSO; Sigma-Aldrich). Before coupling, the chip channel was activated with 1-ethyl-3-(3-dimethylaminopropyl) carbodiimide (EDC, GE Healthcare) and N-hydroxysuccinimide (NHS, GE Healthcare) at a flow rate of 10 μL/min for 60 s. After that, the recombinantly expressed and purified SOAT1 protein was diluted with 10 mmol/L sodium acetate buffer (pH 5.0) to about 50 mg/mL. The flow rate for SOAT1 protein solution was 10 μL/min, and the injection time lasted for 120 s. The protein coupling volume of the channel was about 8000 RU, and the channel was sealed with ethanolamine at a flow rate of 10 μL/min for 120 s. For molecular fragment library screening, the concentration of each compound was diluted to 100 nmol/L with running buffer in a 96-well measuring plate, and then passed through the CM5 chip coupled to the target protein at a flow rate of 30 μL/min for 180 s. After specifying a sample, the chip was regenerated with NaOH at a flow rate of 30 μL/min for 60 s. All raw datum were recorded and stored. When determining the protein–ligand interaction affinity KD value, each compound was diluted 11 times from 20 μM to 0.0195 nM, and the small molecules were passed through the chip coupled to the target protein from low concentration to high concentration. The flow rate was 30 μL/min and the duration was 180 s. After each concentration point flowed through, the chip was regenerated with NaOH at a flow rate of 30 μL/min for 60 s, and then recorded and saved the data in real time. Molecular weight adjustment and solvent correction were used simultaneously to remove non-specific binding and signal drift molecular effects. Finally, all data processing was performed in Biacore T200 analysis software (GE Healthcare).

### Cell viability assay

In order to determine the effect of the compound on the viability of liver cancer cells, the CCK-8 kit (Sigma-Aldrich) was used to detect the cell viability. HepG2, Hep3B, and Huh7 liver cancer cells were cultured in a 96-well plate, using DMEM containing 10% FBS, 100 μg/mL penicillin, and 100 μg/mL streptomycin, in a 37 °C, 5% CO_2_ incubator 12 h, the concentration of a single compound was diluted 10 times from 20 μM, and each concentration point was repeated 3 times. The diluted compound was added to the serum-free medium to treat the cells for 48 h, and then the cells were evaluated with the CCK-8 kit for activity, referring to the instruction manual for the experimental method. After the Tecan microplate reader detects all the absorbance values, the IC_50_ of the drug to the cells is calculated according to the concentration-absorbance value in GraphPad (GraphPad Prism 8.2.1, GraphPad).

### Cell proliferation assay

The cell proliferation experiment was carried out with xCELLigence RTCA DP (Roche), and the E-plate-16 cell detection plate (Roche) was used for cell culture and detection. Specifically, the HepG2, Hep3B, and Huh7 cells are first made into a cell suspension and counted, and the cell concentration is diluted to 8 × 10^4^ cells/L for later use. After adding 50 µL of medium to the wells of E-plate-16 for baseline detection, 100 μL of reconciled cell suspension was added to the wells to make the number of cells per well 8000 cells/100 μL. After standing on the clean bench for 30 min at room temperature, the E-plate-16 is put on the RTCA station again for testing. The working program is set to detect the cell index once every 15 min, record the cell growth process, pause the program after culturing for 3–4 h, add the prepared drugs to it (the concentration of each drug refers to the IC_50_ value of the cell viability determination), and continue the cultivation and observation. After adding the drug, the program is set to detect the cell index once in 15 min and continuously observe and record for 120 h. The cell growth index-time curve was finally drawn in GraphPad Prism 8.2.1.

### Cell migration assay

The effect of the drug on the migration of liver cancer cells was performed using a classic transwell migration assay. HepG2, Hep3B, and Huh7 cells in logarithmic growth phase were taken, and the number of cells was adjusted to 1 × 10^6^ with serum-free DMEM medium. Two hundred microliters of cells was inoculated into the upper chamber of transwell (Millipore). Six hundred microliters of DMEM medium containing 20% FBS was added in the chamber. After culturing in a 37 °C, 5% CO_2_ incubator for 12 h, the drug was added to be tested in the upper and lower chambers of the transwell. The drug concentration shall consult the IC_50_ or twice the concentration determined above. After culturing for 24 h, the transwell was removed with a cotton swab. Cells that did not penetrate the chamber were fixed with methanol for 15 min, washed with PBS 3 times, stained with crystal violet for 30 min, and photographed in five different fields of view from the top, bottom, left, and right under a × 100 optical microscope, and the average number of cells was calculated. Inhibition rate of the drug on cell migration = (1-addition group/control group) × 100%.

### Cell cycle assay

In the cell cycle assay, flow cytometry was used to detect the propidium iodide (PI)-stained cells. The cells were fixed (approximately 2 × 10^6^) in ice-cold buffer with 70% ethanol. Then, the cells were washed and resuspended with PBS. Finally, a reaction solution with 25 mg/mL PI (CWBIO, Beijing, China) and 50 mg/mL RNase A was added to the samples and incubated in the dark for 30 min at 37 °C to stain DNA. The fluorescence emitted from the PI-DNA of individual cells was measured using a BD LSRFortessa (BD, USA) FACS flow cytometer.

### Colony formation assay

HepG2 and Hep3B cells were seeded in a 6-well plate at a density of 1 × 10^3^ cells/well and incubated for 24 h. Cells were treated with nevanimibe (5 μM) and nilotinib (500 nM), and DMSO as a control. Cells were incubated for 8 days. The medium was carefully removed, and cells were washed with PBS and fixed with 100% methanol for 30 min. After removing methanol, cells were stained with 0.5% (w/v) crystal violet for 30 min and washed with tap water. The plate was dried at 25 °C and images were captured. The stained area was automatically measured using ImageJ. This experiment was independently performed 3 times. The area of the colony was calculated as a percentage of the total area of the well.

### Xenograft model

In this study, human HepG2 and murine Hepa1-6 hepatocelluar carcinoma cells were used to in our murine xenotransplant tumor model, and immunodeficiency NOD-SCID mice and normal C57BL/6 J mice (Beijing Vital River Laboratory Animal Technology) were used to model cell-derived xenografts. We resuspended 5 × 10^6^ cells in 200 μl sterile PBS and injected the resuspended cells into the right flank of each NOD-SCID or C57BL/6 J (female, 4–5 weeks old). Once the tumor reached 100 mm^3^, the mice were randomized to groups. In this experiment, the efficacy of nilotinib and sorafenib was evaluated at the same time, and a placebo control group (*n* = 6 for each model) was set up. The dosage of the three drugs was 20 mg/kg/day, and the drugs were administered to the mice via intrapulmonary injection. The administration was expected to continue for 4 weeks, and the tumor size was measured every other day. The mice were killed by spinal dislocation. During the experiment and when assessing the impact, the investigator was unaware of the group medication. The animals were housed in the pathogen-free mouse facility at the National Protein Science Center (Beijing, China) of the Animal Center. According to our Institutional Animal Care and Use Committee (IACUC) protocol, the maximum tumor size of the CDX mouse model is not to exceed 20 mm in any size. All guidelines were followed in this study. Vernier calipers were used to measure tumor size. The following formula was used to calculate the tumor volume in mm^3^: tumor volume = 0.5 × length × (width)^2^.

### Preparation of the proteome and metabolome samples

For omics samples, HepG2 cells grown in log phase were used for experiments. After the cells were cultured at 37 °C and 5% CO_2_ for 12 h, two compounds of nevanimibe and nilotinib were added to each dish (dosing refer to the aforementioned IC_50_), and the control group was equal to 1/1000 One DMSO, and then continue to incubate for 12 h. For proteome analysis, the cells were cultured in 75-cm^2^ plates. Each sample provided 5 × 10e^6^ cells, and each group was repeated three times. In the metabolism analysis, the cells were cultured in 150-cm^2^ plates. The number of cells in each sample was 1 × 10e^7^, and each group was repeated six times. Bio-Rad TC20TM (Bio-Rad) automatic cell counter was used to count all cells.

### Proteome experimental procedures

According to the following experimental conditions, HepG2 cells were divided into three groups: (1) nevanimibe treatment, (2) nilotinib treatment, and (3) DMSO treatment. Each group contains three biological copies. Protein extraction, digestion, peptide separation, and liquid chromatography tandem mass spectrometry (LC–MS/MS) detection methods were roughly the same as our previous research methods. Briefly, the sample was lysed with lysis buffer ultrasonically and then centrifuged at 12,000 × *g* for 10 min at 4 °C to remove cell debris. The supernatant was transferred to a new centrifuge tube, and the protein concentration was determined via a bicinchoninic acid (BCA) Protein Assay Kit (Thermo Fisher Scientific). The same amount of protein in each sample was enzymatically digested, and the peptides digested by trypsin were desalted with Phenomenex and then freeze-dried in vacuo. The peptides were dissolved in the mobile phase A of liquid chromatography (0.1% (v/v) formic acid aqueous solution) and then separated using the nanoflow high-performance liquid chromatograph (HPLC) instrument (Easy nLC1000 System, Thermo Fisher) coupled to an Orbitrap Fusion mass spectrometer (Thermo Fisher) with a nanoelectrospray ion source (Thermo Fisher). Mobile phase A is an aqueous solution containing 0.1% formic acid and 2% acetonitrile; buffer B is an aqueous solution containing 0.1% formic acid and 90% acetonitrile. The mass spectra data were searched using ProteomeDiscover 2.0 and MaxQuant 1.6.1.0 and matched in the SwissProt human proteome database. The matching also used the anti-database to eliminate the false positive rate (FDR) caused by random matching. The protease was set to Trypsin/P, the minimum length of the peptide was set to 7 amino acid residues, the maximum missing cleavage sites was set to 2, and the maximum number of charges was set to 5. The maximum tolerable mass error of the primary precursor ion was set to 10 ppm, and the maximum tolerable mass error of the secondary product ion was set to 0.02 Da. The fixed modification was set to cysteine alkylation, and the variable modification was set to methionine oxidation. The peptide score was greater than 20 points. We also used 1% glyco-diosgenin (GDN) or 5% CHAPS for better extraction of membrane protein during protein extraction.

### Metabolome experimental procedures

Total metabolites were extracted from each group using approximately 1 × 10^7^ cells in 1 ml of MTBE (methyl tert-butyl ether, Sigma-Aldrich) methanol aqueous solution (7:2:1, v/v). First, 100 μL of water was added to the washed cells to resuspend the cells. After mixing, 200 μL of methanol solution was added. The samples were then placed in a shaker and vortexed for 3 min. Then 700 μL of MTBE was added, and the samples were vortexed for 3 min. The cells were then disrupted via ultrasound at 4 °C, 200 W power for 5 min. The program was set to ultrasound for 1 s and pause for 2 s. Finally, the samples were incubated at 25 ℃ for 1 h, centrifuged at 13,000 × *g* for 15 min, and then the supernatant was transferred to a new EP tube. The samples were vacuum-dried at 45 °C to remove organic solvents, and then placed in a freeze dryer to remove the remaining moisture. For LC–MS detection, the samples were dissolved with MTBE methanol aqueous solution (7:2:1, v/v). Samples were analyzed by LC–MS (a UPLC I-class/VION IMS QTOF Mass spectrometry system, Waters). The samples were separated using a C18 reverse chromatographic column (3 μm, Durashell, Agela Technologies). The column temperature was 20 °C, and the flow rate was 0.3 mL/min. The mobile phase of composition A was 10 mM ammonium acetate acetonitrile aqueous solution (acetonitrile: water = 6:4, v/v), and B was 10 mM ammonium acetate acetonitrile isopropanol solution (acetonitrile: isopropanol = 1:9, v/v). The gradient elution procedure was as follows: 0–7 min, B was maintained at 30%; 7–25 min, B changed linearly from 30 to 100%; 25.1–30 min, B was maintained at 30%. The sample was placed in the autosampler at 10 °C during the entire analysis. In order to avoid the influence caused by the fluctuation of the detection signal of the instrument, a random sequence was adopted to carry out continuous analysis of the sample. In the sample queue, one QC sample was set every eight experimental samples in order to monitor and evaluate the stability of the system and the reliability of experimental data. Mass spectrometry conditions were detected using electrospray ionization (ESI) positive ion and negative ion modes. The ESI positive source conditions were as follows: heater temperature 300 °C; spray voltage 3.0 kV; capillary temperature 350 °C; S-lens RF level 50%; MS1 scan ranges 50–1500. The ESI negative source conditions were as follows: heater temperature 300 °C; spray voltage 2.5 kV; capillary temp 350 °C; S-lens RF level 60%; MS1 scan ranges 50–1500. The mass-to-charge ratios of lipid molecules and lipid fragments were collected according to the following method: 10 slice patterns (MS2 scan, HCD) were collected after each full scan. MS1 had a resolution of 70,000 at m/z 200, and MS2 had a resolution of 17,500 at m/z 200. The raw data were collected by MassLynx software (MassLynx 4.2, Watres), and then Progenesis QI (Progenesis QI 2.4, Waters), MS-DIAL (Free down load on http://prime.psc.riken.jp/), and Unifi (Unifi 2.0, Waters) analysis software were used for non-targeting and targeted identification. The raw data of LC–MS/MS were for peak alignments, retention time correction, and peak area extraction. The alignment of MS1 and MS2 tolerance was set to 0.001 Da, and the retention time tolerance was set to 0.02 min. Metabolite structure identification used accurate mass matching (< 10 ppm) and secondary spectrum matching methods to retrieve self-built databases from the laboratory. SIMCA-P (SIMCA 14.1, Umetrics) and GraphPad software were used for data statistics and analysis. R_studio (Free down load on https://www.rstudio.com/) was used to perform correlation analysis and mapping.

### SDS-PAGE and western blot analysis

Proteins were extracted in 25 mM Tris, pH 8.0, 150 mM NaCl, and 3% (w/v) CHAPS buffer. The cells were lysed by sonication on ice for 5 min (sonication for 3 s, off for 2 s; power 60 W), centrifuged at 12,000 × *g* for 15 min at 4 °C, and the supernatant was collected. The protein lysates were quantified with a bicinchoninic acid (BCA) assay and then resolved via SDS-PAGE gel electrophoresis. SDS-PAGE was performed on a 10% SDS-PAGE gel 90 min at 160 V, and then bromophenol blue staining was performed. Photographs were recorded after destaining with acetic acid–methanol-water solution. Western blot analysis was performed on unstained SDS-PAGE gels transferred to nitrocellulose membranes. Membranes were then blocked with 5% non-fat milk in Tris-buffered saline for 1 h at 25 ℃. After blocking, membranes were incubated 12 h at 4 °C with the following primary antibodies: rabbit anti-SOAT1 polyclonal antibody (ABN66, Merck) and rabbit anti-β-actin monoclonal antibody (6609, Proteintech). Membranes were then washed and incubated with secondary antibodies for 1 h at 25 ℃. Protein bands were visualized using enhanced chemiluminescent western blotting substrate.

### Multicolor flow cytometry analysis

To analyze the effect of the SOAT1-targeting compounds on the tumor immunophenotype, the tumors from mice 28 days after dosing were taken down, ground into a single-cell suspension, and resuspended in a centrifuge tube with Hanks’ balanced salt solution (HBSS, Gibco), centrifuged at 2000 rpm for 4 min, and the supernatant was removed. Cells were then digested with collagenases 2 and collagenase 4 to remove connective tissue and collagen components. Finally, the immune cell population was enriched with 35% percoll. We used an anti-CD45-APC/cy7 antibody (103,115, Biolegend), anti-CD11b-APC antibody (K009928M, Solarbio), anti-CD3-BV510 antibody (100,233, BD Biosciences), anti-CD19b-FITC antibody (53,343, Cell Signaling Technology), anti-F4/80-PE antibody (64763S, Cell Signaling Technology), anti-Ly6G-Alexa fluor 700 antibody (127,622, Biolegend), anti-CD8-PerPC/cy5.5 antibody (100,733, Biolegend), and anti-CD49- BV421 antibody (740,030, BD Biosciences) for staining. The samples were collected by the BD LSR Fortessa flow cytometer (BD Biosciences). We then utilized FlowJo software (Free download on https://www.flowjo.com/) to analyze the data. The fluorescence-activated cell sorter (FACS) gating/sequencing strategy is shown in Supplementary Figure S6.

### Bioinformatics analysis

In this study, the UniProt-GOA database (http://www.ebi.ac.uk/GOA/) and the Kyoto Encyclopedia of Genes and Genomes (KEGG) database (https://www.kegg.jp/) Gene Ontology (GO) annotations and metabolic pathway annotations are provided. Volcano and Venn diagrams were used to identify and classify differential proteins or metabolites. All differentially expressed proteins or metabolites were imported into R_studio to search for relevant pathways. The outputs of the pathways were automatically classified into rank categories and were considered significant when the adjusted *P* value was < 0.05, and a *P* < 0.01 was considered very significant. GO analyzes the biological processes, cellular components, and molecular functions of dysregulated proteins or metabolites. Differential proteins or metabolites of each signaling pathway were marked using the online website KEGG, and the final images were visualized in Cytoscape 7.1 [18]. R_studio was used to draw the various volcano maps, Venn diagrams, scatter plots, and correlation heat maps.

### Qantification and statistical analysis

For detailed analysis of statistical results, please refer to each method. The *P* values appearing in this report were calculated by two-tailed Student’s *t* test. A *P* < 0.05 was considered significant, and a *P* < 0.01 was considered very significant. Relative standard deviation (RSD) was utilized to express the accuracy of the analytical test results. The smaller the value, the better the repeatability. The false discovery rate (FDR) is the expected value of the number of false rejections, as a percentage of all rejected hypotheses. As a control index for the test hypothesis error rate, the control value was selected according to the need, and the value of the traditional hypothesis test was usually set to 0.05. In this study, all biochemical analyses were performed independently at least three times. GraphPad Prism 8.21 was utilized to analyze the data. A Pearson correlation analysis was used to measure the linear correlation of the data. A volcano diagram was used to display the results of differential expression analysis, and a Venn diagram was used to display the logical connection between different groups (sets). GO enrichment analysis and KEGG pathway analysis are based on online tools (such as KEGG mapper and string) and offline tools (such as Cytoscape 3.7). Microsoft Excel was used for other non-computational analyses (Microsoft Excel 2019).

### Data and code availability

The protein MS data were deposited in iProX [19] (an official member of ProteomeXchange consortium) as an attachment file (http://www.iprox.org) with the project ID IPX0003944000. The metabolomics data reported in this paper were deposited in MetaboLights [20] as an attachment file (https://www.ebi.ac.uk/metabolights/MTBLS4088) with the Project ID MTBLS4088.

## Results

### SOAT1-based drug screening initially obtained three candidate compounds

**Fig. 1:**
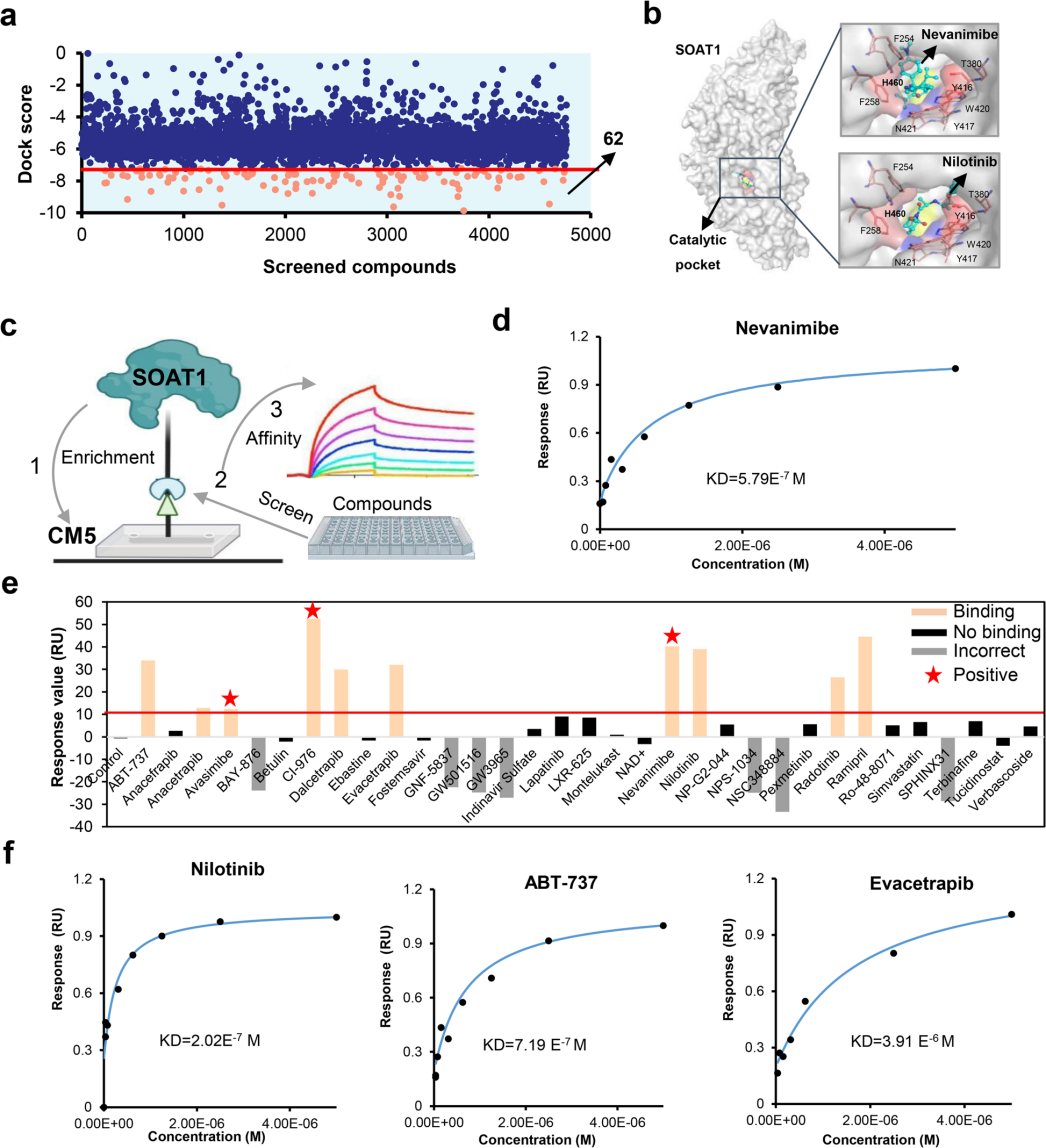
Drug screening based on SOAT1 protein. **a** Structure-based SOAT1 protein virtual screening yielded 62 potential compounds (in orange). The top 200 compounds were selected from the screening results of each software and were screened by at least three other software platforms. All results were compared with avasimibe, with a relative standard deviation (RSD) < 15%. **b** Both the SOAT1 receptor and control compound (nilotinib) from the screened compounds bound at the catalytic site as compared with the positive control nevanimibe. **c** Drug screening schematic of the SOAT1 protein by surface plasmon resonance (SPR). **d** The KD of the known co-crystal ligands (nevanimibe) of SOAT1 was calculated by SPR to confirm the reliability of the system. **e** SPR fragment library screening verified 33 virtual screening results. Among them, there were 10 compounds with a response unit (RU) > 10 (orange histogram), including three positive controls (red star), 16 compounds that did not bind to the protein (black histogram), and 7 other compounds had signal errors (gray histogram). **f** The KD value of the screened ligands was calculated by SPR

Previously, we found that SOAT1 protein is closely related to the survival of the most malignant hepatocelluar carcinoma patients, promising to become a potential target for hepatocelluar carcinoma therapy [8]. In this study, we developed a virtual screening integrated affinity screening technology based on SOAT1 protein to rapidly discover target protein ligands. The overall workflow started with the high-resolution crystal structure of human SOAT1 homodimer and tetramer (PDB code: 6VUM and 6L47) [12, 13] (Figure S1a). The catalytic cavity at the His460 residue of SOAT1, which is considered to be the catalytic site for the synthesis of cholesterol esters between cholesterol and acetyl-CoA, was defined as the screening target site. Molecular docking was implemented using four different tools ensembledocking (AutoDock 4.2, SYBYL 2.0, and standard precision (SP) and extra precision (XP) modes in Glide) to improve the accuracy and hit rate of the virtual screening. All 31,276 compounds in the FDA libraries were utilized, and as a result, 62 total compounds, ranked by all four docking tools with a score higher than that of the positive control, were identified as putative ligands (Fig. 1a, and table S1, full of docking results shown in a separate additional file 1). Further high-precision molecular docking analysis demonstrated that the screened compounds (nilotinib as a representative) and the crystal compound of nevanimibe both bound to the catalytic pocket of SOAT1 protein (Fig. 1b).

SOAT1-based affinity screening was performed to verify the virtual screening putative ligands. The full-length cDNA of human SOAT1 (NCBI ID: 6646) was cloned into the pCAG vector with a carboxy-terminal 6 × His-tag (Figure S1b,c and d). Protein was expressed in HEK293F cells, and after a series of protein purification steps, a stable dimer form of SOAT1 protein was obtained (Figure S1e and f). The purified protein was fixed on a CM5 chip for affinity screening and verification by surface plasmon resonance (SPR). The general process is shown in Fig. 1c. First, analysis of the positive controls nevanimibe showed that suitable binding to SOAT1 protein was exhibited, which indicated that the screening system was robust (Fig. 1d). Then the 33 highest scoring and easy-to-obtain compounds (about half of the 62 candidate compounds in the virtual screening) were further identified by SPR fragment library screening. From these 33, 10 compounds were determined to bind to SOAT1 (including three positive controls: avasimibe, nevanimibe, and CI-976) (Fig. 1e, orange column), 16 compounds had almost no binding to SOAT1 protein (Fig. 1e, black column), and 7 compounds could not be accurately determined by SPR due to the characteristics of the molecules themselves (Fig. 1e, gray column). These results indicated that even though the screening rules were strictly formulated during the virtual screening process, false positives still exist. Finally, these false positives were eliminated through affinity screening.

Subsequently, we further determined the affinity index KD value of the above 10 compounds with SOAT1 through SPR. The results showed that three compounds, nilotinib, ABT-737, and evacetrapib, exhibited a KD value of 2.02E^−7^ M, 7.19 E^−7^ M, and 3.91 E^−6^ M, respectively (Fig. 1f). Nilotinib had lower KD values for SOAT1 protein as compared with the positive controls nevanimibe, indicating the potential they have as SOAT1-targeted drugs. In summary, we developed a virtual screening and integrated affinity screening strategy for the rapid and efficient discovery of ligands for SOAT1 protein and ultimately obtained three candidate compounds that targeted SOAT1 protein.

### Compounds targeting SOAT1 protein showed outstanding antitumor activity in vitro and in vivo

**Fig. 2:**
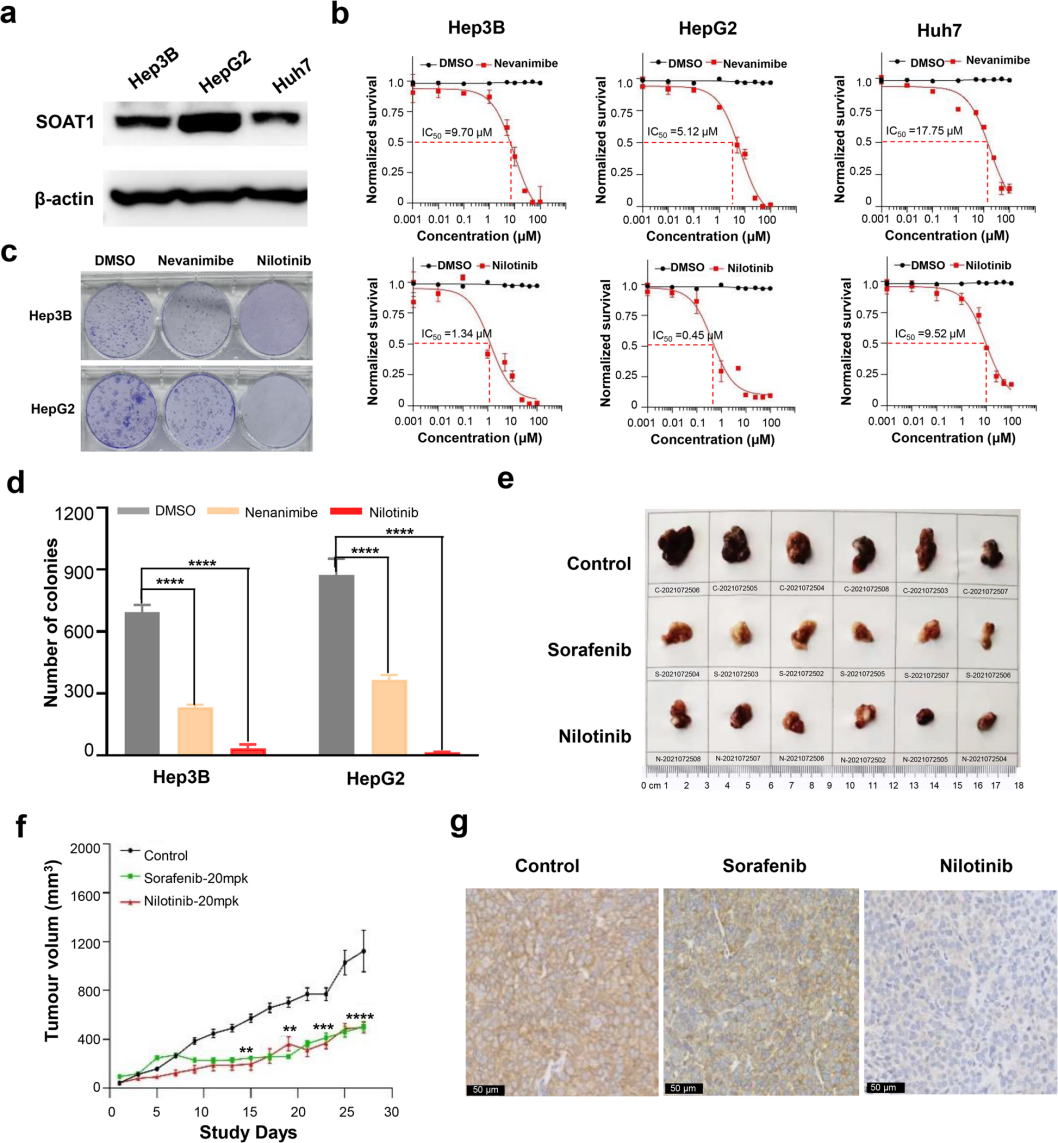
Verification of the in vitro and in vivo activity of the identified compounds. **a** Relative expression of SOAT1 in different liver cancer cell lines (*n* = 3). **b** Dose–response curves of hepatocelluar carcinoma cell lines to nilotinib treatment compared with the positive control (nevanimibe), with an endpoint measurement at 48 h (*n* = 3). **c, d** Colony formation assay in Hep3B and HepG2 cells. The area of the colony was analyzed using the ImageJ software and plotted using GraphPad Prism 8.21 (*n* = 3). Graphs show mean ± SD. **e** Images of the tumor volume in mice treated with the nilotinib, sorafenib, and control for 28 days. The positive control is sorafenib and was compared with placebo controls. **f** Tumor growth curve of tumor xenograft mice treated with nilotinib (20 mg/kg/day), sorafenib (20 mg/kg/day), and controls on the indicated days (*n* = 6 mice per group). The circle, triangle, and box denote the average volume of tumors (± SEM). *P* values were calculated by the two-sided log-rank test. **g** Immunohistochemical staining of SOAT1 in tumors after nilotinib, sorafenib, and control (scale bar = 50 μm)

The expression level of SOAT1 was correlated with the overall survival rate of hepatocyte patients in our proteomics and a tissue microarray (TMA) cohort and the Cancer Genome Atlas (TCGA) data set [21]. The higher the level of SOAT1 expression, the lower the overall survival of patients. In our previous study, we have also demonstrated that knockdown of the SOAT1 gene can significantly inhibit the growth of hepatocellular carcinoma cell lines [8]. These results indicated that compounds targeting SOAT1 have the potential to inhibit the growth of hepatocelluar carcinoma. In order to determine the effect of the SOAT1-targeting compounds on hepatocelluar carcinoma, we selected three different hepatocelluar carcinoma cell lines, Hep3B, HepG2, and Huh7, for drug activity testing. Western blot results showed the relative expression of SOAT1 in HepG2 cells to be higher than that in Hep3B cells and Huh7 cells (Fig. 2a, original, uncropped blots shown in additional file 2). The representative compound nilotinib was compared with the positive controls nevanimibe. Both cell migration and cell proliferation results showed that the two compounds significantly inhibited the migration and proliferation of liver cancer cells at various concentrations, but nilotinib achieved the inhibitory effect at lower concentrations (Fig. 2b and Figure S2a, b). The half-maximal inhibitory concentration (IC_50_) of the two compounds on the three liver cancer cell lines also showed that the IC_50_ value of nilotinib was much lower than that of the positive controls nevanimibe (Fig. 2b). Moreover, the inhibitory effect of the compounds on cell lines with high SOAT1 expression was better than that on cell lines with low SOAT1 expression. This indicated that compounds targeting SOAT1 have a better therapeutic effect on patients with high SOAT1 expression, which is also consistent with our previous results. Finally, the results of colony formation and cell cycle experiments showed that nevanimibe and nilotinib can significantly arrest cells in the early DNA synthesis stage and blocked DNA replication phase (G1 phase and S phase) (Figure S2c) and inhibited colony formation of hepatocellular carcinoma cells (Fig. 2c, d).

To further determine the therapeutic effect of the compounds on hepatocelluar carcinoma, we extended the evaluation of the efficacy to a murine tumor xenograft model. First, we observed the effect of the compound on the physiological state of the target mice (NOD-SCID). The experimental results showed that treatment with nilotinib and positive control drug sorafenib for 4 weeks had no effect on the body weight of the xenograft mice (Figure S2d). Subsequently, nilotinib was injected intraperitoneally into HepG2 cell-derived xenograft mice at a dose of 20 mg/kg/day (*n* = 6). A placebo was used as a blank control, and the first-line liver cancer treatment drug sorafenib was used as the positive control. The administration was continued for 4 weeks, and the tumor growth was observed and recorded. As shown in Fig. 2e, after 4 weeks of continuous administration, the tumor volume in the nilotinib-treated groups was significantly smaller than the blank control group and comparable to the positive control group. The tumor growth curve also showed that the tumor growth rate of the nilotinib-treated groups and the positive control group was significantly lower than that of the blank control group (Fig. 2f). Immunohistochemical staining of SOAT1 in tumors after administration of the compounds showed that SOAT1 protein in the tumor tissues of mice was significantly lower than that of the control group (Fig. 2g). These results not only confirmed that compounds targeting SOAT1 can significantly inhibit the growth of hepatocelluar carcinoma, but also indicated that they may have clinical potential as a new treatment for hepatocelluar carcinoma.

### Proteomics analysis revealed that compounds targeting SOAT1 restored cholesterol metabolism

**Fig. 3:**
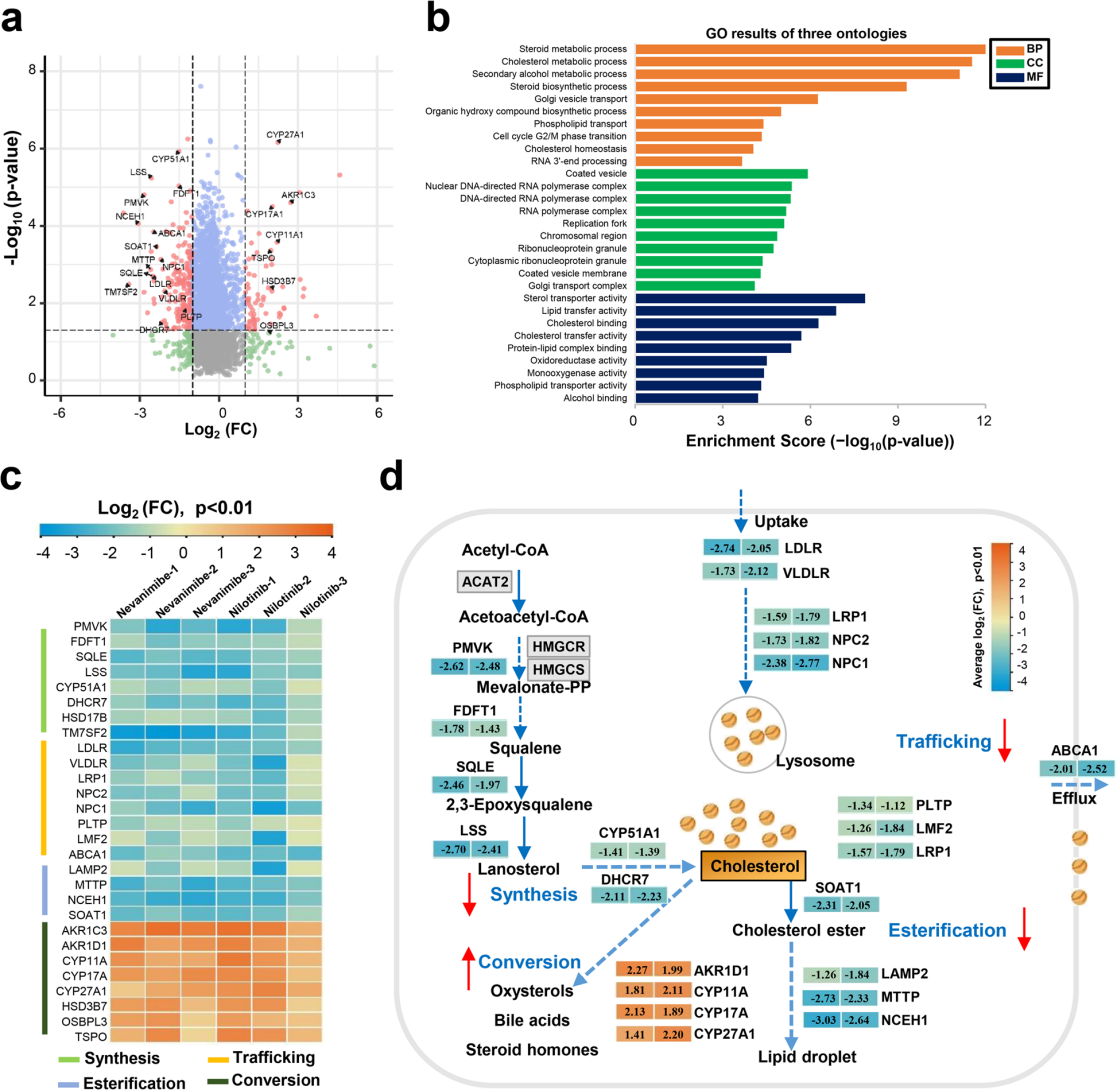
Changes in cholesterol metabolism pathway proteins after drug treatment. **a** Proteomic data were analyzed by volcano plots, with the *x*-axis representing the log_2_ (FC) (sample/control), and the *y*-axis representing the − log_10_ *P* value. Red dots indicate significantly different proteins regulated by SOAT1-targeted compounds; gray, blue, and green dots indicate proteins that are meaningless or poor qualitative data. Significant proteins are marked by boxes. **b** Gene Ontology (GO) analyzes the biological process, cellular component, and molecular function of the dysregulated proteins. *P* values were corrected by the Benjamini-Hochberg (BH) algorithm and significant calls were made based on a BH adjusted *P* value < 0.01. **c** The cluster heat map of the dysregulated cholesterol metabolism proteins after treatment with SOAT1-targeted compounds. **d** Biological insight into the cholesterol metabolism reprogramming after treatment with SOAT1-targeted compounds. The alteration score of each protein is depicted as log ratios (fold change, expressed as log2 (ratio of average protein abundance in each treatment proteomic results versus the control group)). Rectangular color bars indicate upregulated and downregulated proteins

In many tumor patients, cholesterol synthesis and uptake are significantly upregulated, with significant accumulation of cholesterol derivatives, such as cholesterol esters and oxidized cholesterol [22]. However, the underlying molecular mechanism of cholesterol metabolism in liver cancer cells is still not well understood. Here, we performed proteomic analysis on the liver cancer cell line HepG2 treated with SOAT1-targeting compounds and tried to clarify the connection between cholesterol homeostasis regulation and tumors and the role of cholesterol homeostasis regulatory proteins in tumorigenesis and development. In order to acquire a comprehensive view of the proteome in liver cancer cells after treatment with SOAT1-targeting compounds, we simultaneously assessed HepG2 cells treated with nevanimibe and nilotinib for 12 h to detect protein changes by liquid chromatography tandem mass spectrometry (LC–MS/MS), and 0.1% DMSO was used as a control (*n* = 3) (Figure S3a). In general, the data of all groups were highly repeatable with a biological repetition rate > 95%, and the correlation coefficient of all samples was > 85% (Figure S3b). In total, 3960 proteins were quantified from 7307 identified proteins (Figure S3c, full of proteome data shown in separate additional file 3). After correcting for multiple testing by setting the *P* value at 0.01, false discovery rate (FDR) at 0.05, and fold change > 2, 226 differential proteins (180 downregulated and 48 upregulated) were simultaneously dysregulated in the proteome of two compounds targeting SOAT1 (Fig. 3a and Figure S3d). Then, we submitted all differentially expressed proteins to Gene Ontology (GO) enrichment and Kyoto Encyclopedia of Genes and Genomes (KEGG) pathway annotation. GO and KEGG analyses showed that the dysregulated proteins were related to steroid metabolic processes or cholesterol metabolic processes, and the corresponding molecular functions were also closely related to sterol transporter activity, lipid transfer activity, cholesterol binding, and cholesterol transfer activity (Fig. 3b). These results indicated that the most dysregulated proteins were generally related to cholesterol metabolism. Further research on cholesterol metabolism signaling pathway proteins found 29 cholesterol metabolism–related proteins significantly altered after treatment with SOAT1-targeting compounds (Table S2). Classification and analysis of these differential proteins showed that most of the cholesterol synthesis–related proteins in the cholesterol metabolism signaling pathway, such as phosphomevalonate kinase(PMVK), lanosterol 14-alpha demethylase(CYP51A1), 7-dehydrocholesterol reductase(DHCR7), lanosterol synthase(LSS), and squalene monooxygenase(SQLE), were significantly downregulated. Additionally, cholesterol transport and esterification–related proteins, including NPC1, SOAT1, phospholipid transfer protein (PLTP), and neutral cholesterol ester hydrolase 1(NCEH1), were also significantly downregulated. In contrast, cholesterol conversion–related proteins, such as sterol 26-hydroxylase (CYP27A1), steroid 17-alpha-hydroxylase/17,20 lyase (CYP17A), aldo–keto reductase family 1 member (D1AKR1D1), and translocator protein (TSPO), were significantly upregulated (Fig. 3c). Cholesterol synthesis, esterification, transportation (including uptake and efflux), and conversion are the main biological processes that maintain the stability of cholesterol metabolism [23]. Numerous previous clinical and preclinical studies have shown that successful treatment of tumors can be achieved by interfering with the cholesterol metabolism of tumor cells and immune cells [24]. Our proteomics analysis showed that compounds targeting SOAT1 not only block the production of cholesterol esters, but also inhibit the synthesis and uptake of cholesterol and advance the conversion of cholesterol to bile acids. Thus, SOAT1-targeting compounds systematically restored the cholesterol metabolism in tumor cells (Fig. 3d).

### Metabolomics analysis confirmed that compounds targeting SOAT1 inhibited cholesterol synthesis, esterification, and transport and promoted cholesterol conversion

**Fig. 4:**
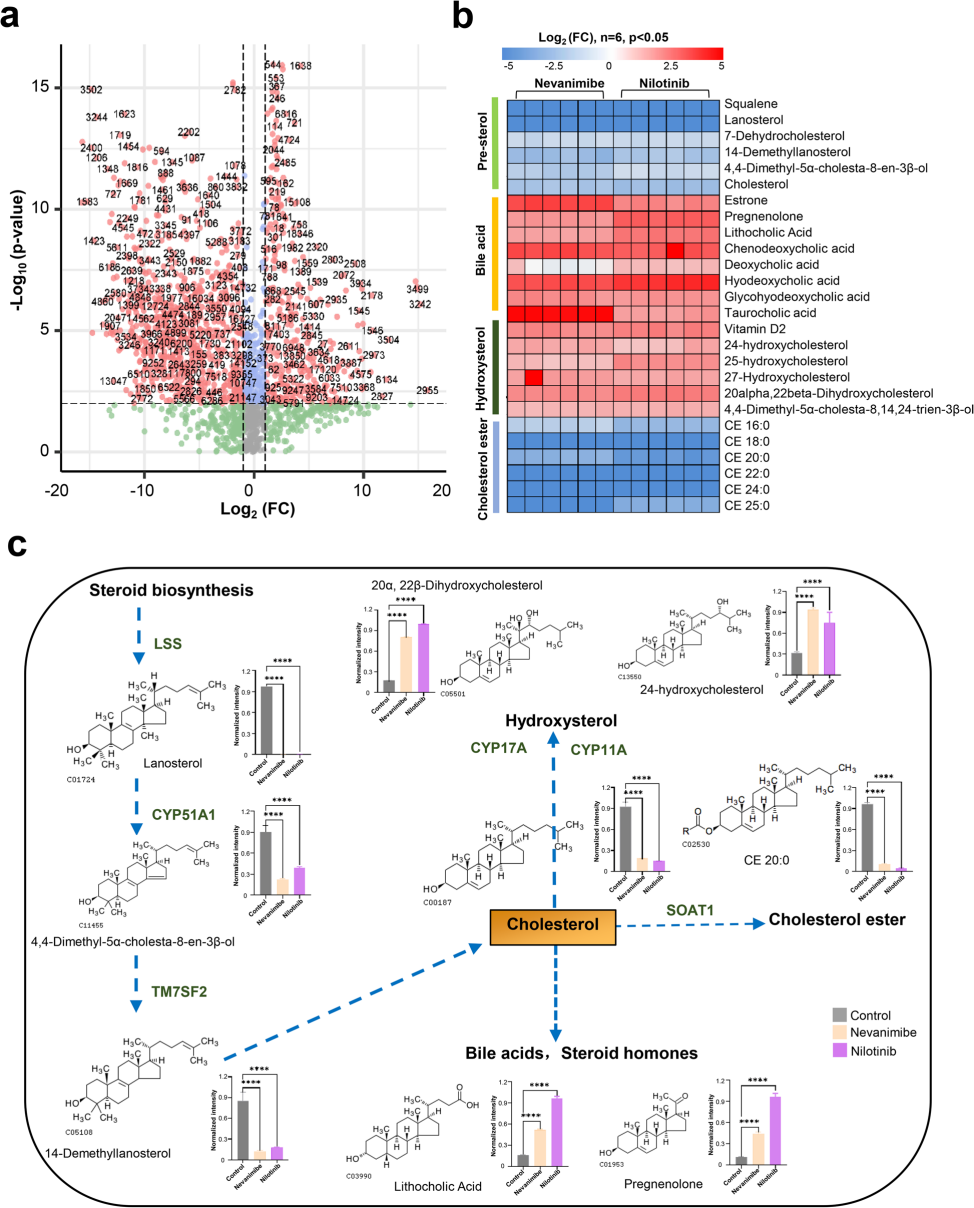
Changes in cholesterol synthesis precursors and metabolic derivatives after drug treatment. **a** The metabolome data were analyzed by volcano plots, with the *x*-axis representing the log2 (FC) (sample/control), and the *y*-axis representing the − log_10_ *P* value. Red dots indicate significantly different metabolites regulated by SOAT1-targeted compound; gray, blue, and green dots indicate proteins that are meaningless or poor qualitative data. **b** Changes in cholesterol metabolites in hepatocelluar carcinoma cell lines after treatment with SOAT1-targeted compounds. The alteration score of each metabolite is depicted as log ratios (fold change, expressed as log_2_ (ratio of metabolite abundance in each treatment proteomics versus the control group)) (*P* < 0.01). **c** Representative metabolite changes in the cholesterol metabolism signaling pathway after treatment with SOAT1-targeted compounds (*n* = 6). Graphs show mean ± SD. *P* values were calculated by the two-sided log-rank test

In order to further clarify the connection between cholesterol metabolism and tumors, we have conducted cell lipid metabolism research. Similar to our proteomics experiment, we simultaneously treated HepG2 cells with two SOAT1-targeting compounds, nevanimibe, and nilotinib, for 12 h, and 0.1% DMSO was used as a control (*n* = 6). The previously reported MTBE (methyl tert-butyl ether) method was used to extract lipid metabolites from tumor cells [25]. These extracted metabolites were then delivered to LC–MS/MS for analysis (Figure S4a). Pearson correlation analysis results were visualized in a correlation heat map, which represented the parallelism of the biological duplication of each sample data (Figure S4b). Overall, 1890 compounds were quantified from 19,671 identified MS/MS spectra, and 662 metabolites were classified (Figure S4c, full of metabolome data shown in separate additional file 4). After correcting for multiple testing by setting the *P* value at 0.01, false discovery rate (FDR) at 0.05 and fold change > 2410 differential metabolites (239 downregulated and 171 upregulated) were detected in the metabolome of nevanimibe and nilotinib (Fig. 4a and Figure S4d). To more accurately identify differential changes in metabolites associated with cholesterol metabolism pathways, we further performed targeted metabolome analysis using metabolite standards. As a result, we found that 26 cholesterol metabolism pathway metabolites changed after administration (Table S3). Heat map cluster analysis showed that hydroxysterol, bile acid, and sterol hormone compounds were significantly upregulated, while pre-sterol and cholesterol ester were significantly downregulated (Fig. 4b). Specifically, hydroxysterol, bile acids, and steroid hormone metabolites, such as 20-α, 22-β-dihydroxycholesterol, 24-hydroxycholesterol, lithocholic acid, and pregnenolone, were significantly increased, while pre-sterol and cholesterol ester metabolites, such as lanosterol, 4,4-dimethyl-5-α-cholesta-8, 14-demethyllanosterol, 18:0 cholesterol ester, and 20:0 cholesterol ester were significantly reduced (Fig. 4c). These results further confirmed that compounds targeting SOAT1 can inhibit cholesterol synthesis, esterification, and transport, and promoted cholesterol conversion.

### Compounds targeting SOAT1 reshaped cholesterol metabolism programming in clinical liver cancer patients

**Fig. 5:**
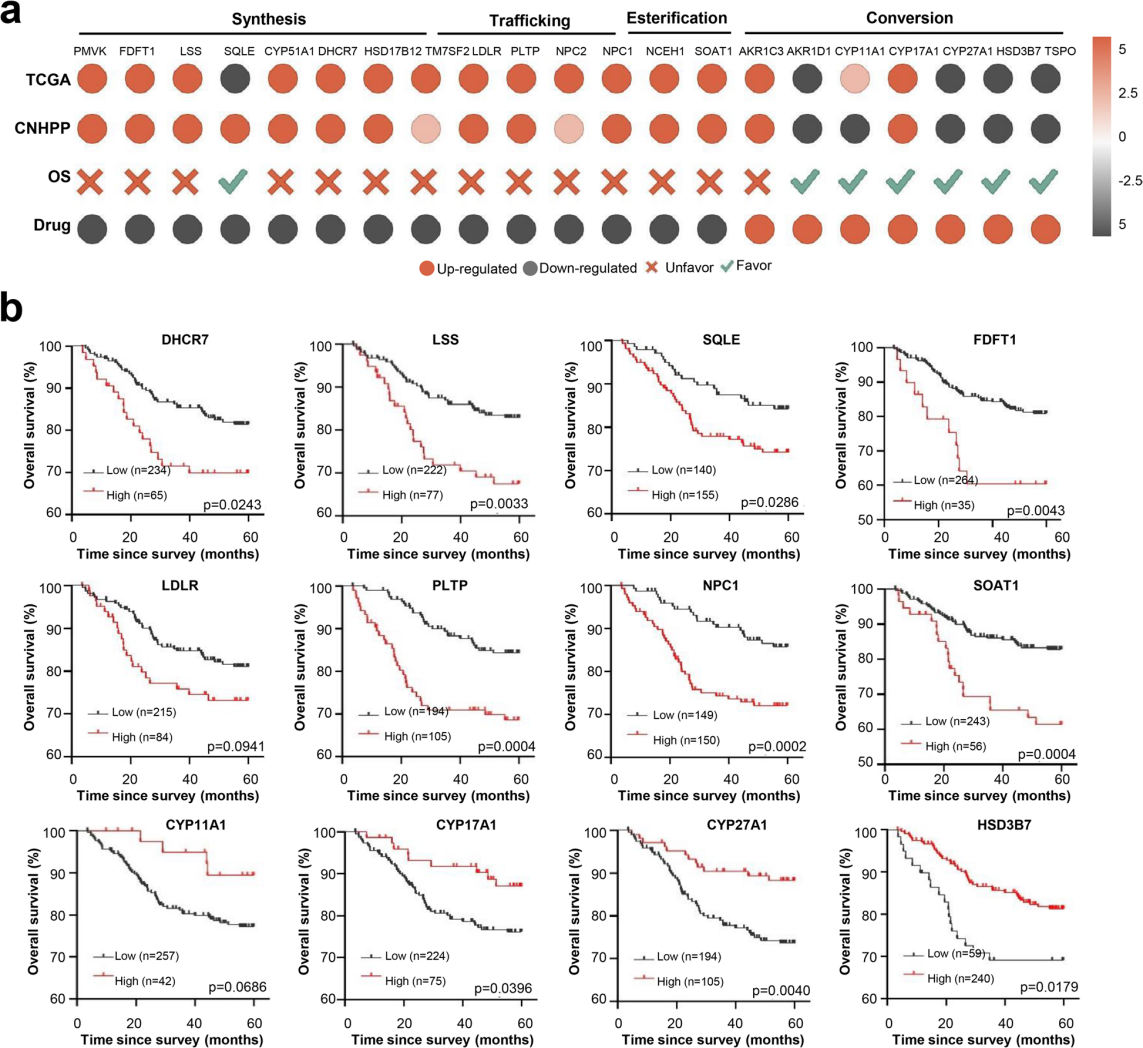
Association analysis of drug proteomics and clinical data of liver cancer patients. **a** Comparative analysis of the drug proteome and clinical liver cancer patient’s proteome and The Cancer Genome Atlas (TCGA) liver cancer patient’s transcriptome data. **b** The relationship between drug-regulated cholesterol dysregulation proteins and the survival of liver cancer patients

Increasing evidence has suggested that cholesterol metabolism programming is associated with an increased risk of recurrence and higher mortality rate in liver cancer patients [26]. In order to explore the relationship between the cholesterol metabolism pathway proteins affected by the identified SOAT1-targeting compounds and the survival of liver cancer patients, we retrospectively compared and analyzed the proteome data of clinical liver cancer patients in the Chinese Human Proteome Project (CNHPP) (http://liver.cnhpp.ncpsb.org/), transcriptome data of TCGA (https://www.genome.gov/Funded-Programs-Projects/Cancer-Genome-Atlas) liver cancer patients, and our proteome data generated in this study. These data indicated that the abovementioned cholesterol metabolism pathway proteins regulated by compounds targeting SOAT1 are expressed opposite to the autologous proteome data and transcriptome data of liver cancer patients, indicating that the regulation of cholesterol metabolism would be reversed and restored in liver cancer patients after treatment with SOAT1-targeting drugs (Fig. 5a). Then we analyzed the survival follow-up information of these patients (Shown in separate additional file 5), and we found that these cholesterol metabolism regulatory proteins are indeed related to the survival of liver cancer patients. The high expression of cholesterol synthesis–related proteins and cholesterol esterification–related proteins are not conducive to the survival of liver cancer patients, while the high expression of cholesterol conversion–related proteins is conducive to the survival of liver cancer patients (Fig. 5b and Figure S5). These results indicated that SOAT1-targeting compounds restore cholesterol metabolism and is a promising treatment to promote the survival of liver cancer patients.

### SOAT1-targeting compounds simultaneously enhance immune cells to inhibit tumor growth

**Fig. 6:**
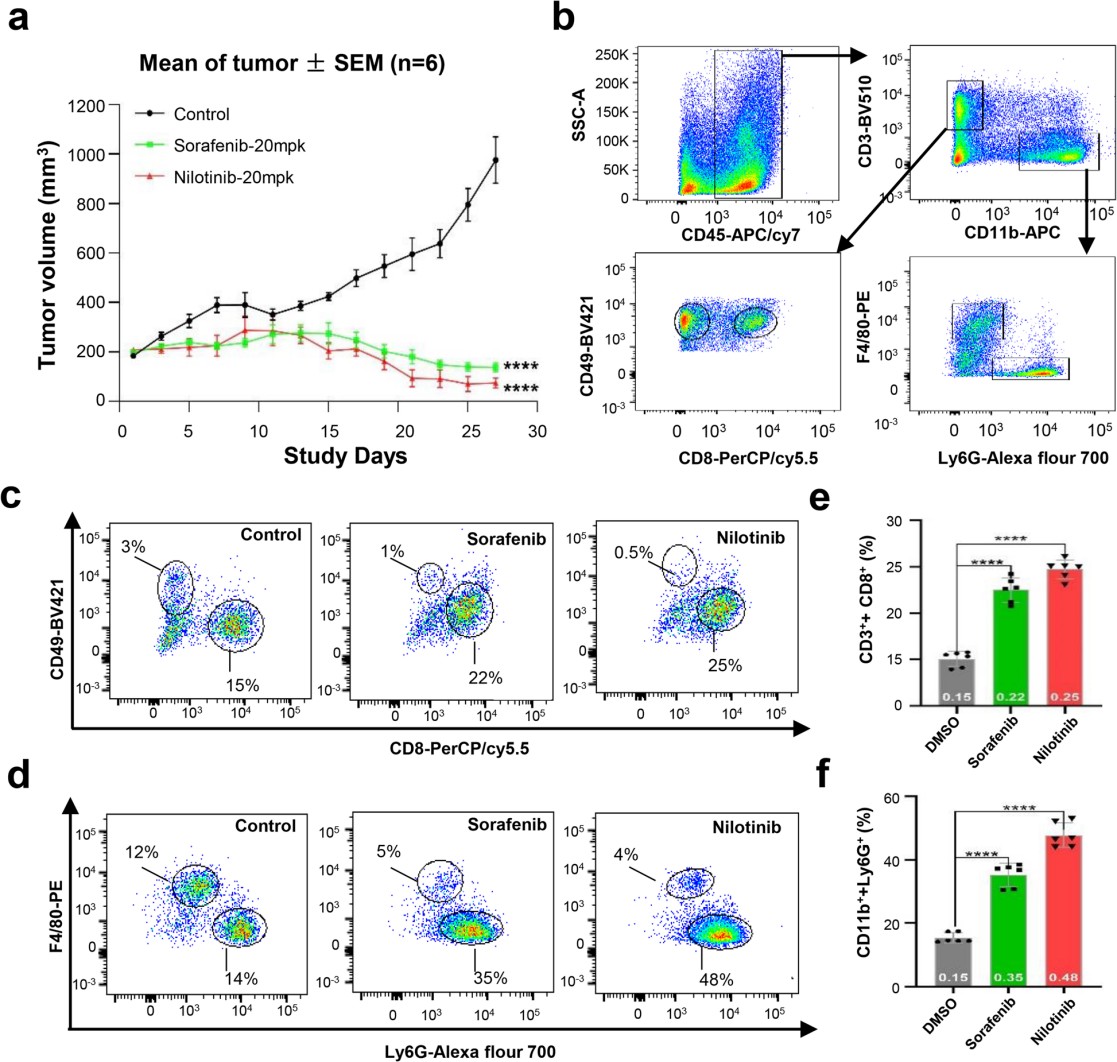
Verification of the in vitro and in vivo activity of the identified compounds. **a** Tumor growth curve of tumor xenografts in C57BL/6 J mice treated with nilotinib (20 mg/kg/day), sorafenib (20 mg/kg/day), and controls on the indicated days (*n* = 6 mice per group). The circle, triangle, and box denote the average volume of tumors (± SEM), and *P* values were calculated by the two-sided log-rank test. **b** Gating based on cell types labeled with different flow cytometry antibodies. SSC-A means cell granularity, CD45 labeled leukocytes, CD3^+^ and CD8^+^ labeled cytotoxic T cells, CD3^+^ and CD49^+^ labeled NK cells, CD11b^+^ and F4/80^+^ labeled macrophages, CD11b^+^ and Ly6G^+^ labeled neutrophils. **c, d** Cytokine production of stimulated tumor xenograft murine CD8^+^, CD49^+^, F4/80^+^, and Ly6G^+^ cells pretreated with nilotinib, sorafenib, or DMSO (*n* = 6). **e, f** Mean SD relative quantity percentage of CD3^+^ + CD8^+^, CD3^+^ + CD49^+^, CD11b^+^ + F4/80^+^, and CD11b^+^ + Ly6G^+^ cells pretreated with nilotinib, sorafenib, or DMSO (*n* = 6). *P* values were calculated by the two-sided log-rank test

A previous study showed that inhibiting cholesterol esterification in T cells by genetic ablation or pharmacological inhibition of SOAT1 significantly improved the CD8^**+**^ T cell antitumor response in mice [10, 11]. The increase in cholesterol levels in the plasma membrane of CD8^**+**^ T cells leads to the accumulation of T cell receptors and enhanced signal transduction, as well as more effective formation of immune synapses [27]. In order to better understand the relationship between SOAT1 inhibitors and immune cell populations and their effects on tumors, we used multicolor flow cytometry to detect changes in immune cell populations after administration of SOAT1-targeting compounds in a murine Hepa1-6 cell xenograft model. We transplanted Hepa1-6 cells into immunocompetent mice (C57BL/6 J) to construct a new tumor xenograft analysis model. After treatment with nilotinib, and positive control drug sorafenib for 4 weeks at a dose of 20 mg/kg/day (*n* = 6), the tumors were harvested and the immune cell population changes were analyzed. As showed in Fig. 6a and Figure S6a, in tumor xenograft model mice with immunity, the compounds targeting SOAT1 had significant tumor suppressor effects. Even 2 weeks after the administration, the tumor’s size had subsided. Immediately afterwards, we take down these tumors and performed multicolor flow cytometry analysis according to the previously described method [28, 29]. CD45-APC/cy7, CD11b-APC, CD19b-FITC, CD3-BV510, F4/80-PE, Ly6G-Alexa fluor 700, CD8-PerPC/cy5.5, and CD49-BV421 were used to label the different types of cells. According to the experimental results, the distribution of tumor cell populations in mice changed significantly after administration (Fig. 6b). Specifically, after drug treatment, the percentage of CD3^+^ + CD8^+^ labeled cells and CD11b^+^ + Ly6G^+^ labeled cells were significantly increased in total CD45 labeled leukocyte cell number, while the percentage of CD3^+^ + CD49^+^ labeled cells and CD11^+^ + F4/80^+^ labeled cells were relatively reduced (Fig. 6c, d). In particular, compared with a previous report [10], not only the proportion of CD3^+^ + CD8^+^ labeled T cells but also CD11b^+^ + Ly6G^+^ labeled neutrophils in tumor tissues of mice treated with nilotinib and sorafenib increased significantly (Fig. 6e, f). These results indicated that the SOAT1-targeting compounds we identified are also able to potentiate the antitumor effects of immune cells.

### Summary of screening SOAT1 targeted compounds and their antitumor mechanisms

**Fig. 7:**
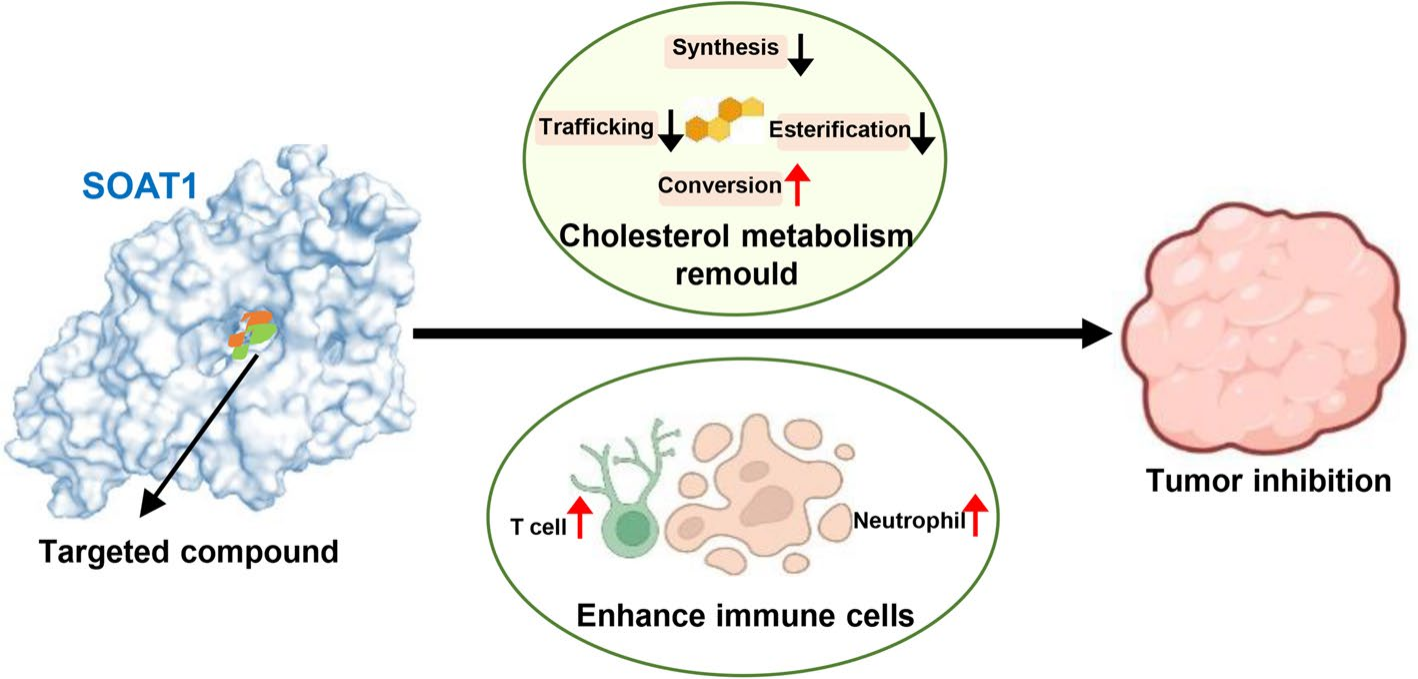
Antitumor mechanism of SOAT1-targeting compounds. Compounds that target SOAT1 protein reprogram tumor cholesterol metabolism, specifically blocking the production of cholesterol ester, inhibiting cholesterol synthesis and transportation, and activating conversion. These compounds also simultaneously enhance immune cells to inhibit tumor growth

Previous studies have reported that cholesterol and its metabolites play an important role in cancer [30, 31]. Recent preclinical studies have shown that blocking cholesterol synthesis and uptake has an inhibitory effect on tumor formation and growth [32]. In this study, we established a high-throughput screening technology based on target protein–ligand interactions through the integration of virtual screening and affinity screening technology. Three compounds nilotinib, ABT-737, and evacetrapib were exhibited optimal binding with SOAT1. In particular, nilotinib displayed a high affinity for SOAT1 protein and significantly inhibited tumor activity both in vitro and in vivo, highlighting their clinical potential in the treatment of liver cancer. Proteome and metabolism studies demonstrated that compounds targeting SOAT1 protein not only directly block the conversion of cholesterol into cholesterol esters by inhibiting the activity of SOAT1 protein, but also reprogram the cholesterol metabolism. To be precise, cholesterol synthesis is inhibited, transportation (including intake and efflux) is blocked, and conversion is activated (derivatization for bile acid, hydroxycholesterol, and sterol hormones). Changes to these substances have been confirmed by various studies to have an inhibitory effect on tumor cells, including improving the immune microenvironment, such as T cells and neutrophils (Fig. 7). It has also been reported that the changes in these cholesterol metabolism signaling pathway proteins are indeed related to the survival of liver cancer patients. In general, we not only established a high-throughput screening technology based on the SOAT1 protein and initially obtained two promising candidates for the treatment of liver cancer, but also systematically revealed that SOAT1-targeting compounds reprogram cholesterol metabolism to remedy hepatocelluar carcinoma.

## Discussion

Screening of antitumor drugs that target cholesterol metabolism programming is a popular area of research, and some target drugs are currently being promoted to clinical or preclinical research [3, 4]. Several drugs, such as HMGCR, MVK, LDLR, and FDPS, have progressed significantly in drug development, but most drugs are still in clinical or preclinical phases of research [33, 34]. Therefore, the discovery of new therapeutic targets that can be developed into effective and tolerable novel drugs for patients with advanced hepatocelluar carcinoma is a long-term, continuous work [35, 36]. In this study, we established a target-based, high-throughput drug screening based on SOAT1 protein that utilizes virtual screening and integrated affinity screening technology. By applying this strategy, we have not only succeeded in identifying SOAT1 protein ligands from tens of thousands of compounds (Fig. 1). We identified nilotinib as SOAT1-targeting compounds, and we further demonstrated via various experiments that these compounds have a high affinity with SOAT1 protein and significantly inhibit tumor activity in vitro and in vivo (Fig. 2). Our research not only established a rapid and efficient target protein–ligand screening technology, but also obtained effective candidate drugs for the targeted therapy of hepatocelluar carcinoma. This study provided new insights for the precise treatment of hepatocelluar carcinoma.

Tumor cell metabolic reprogramming is considered to be one of the 10 characteristics of tumors, and cholesterol metabolic reprogramming of tumor cells has been shown to play an important role in tumorigenesis [37]. However, the underlying mechanism of cholesterol metabolism reprogramming in tumor cells and drugs that target cholesterol metabolic reprogramming in tumors is still unknown [26, 38]. In this study, we applied proteomics and integrated metabolomics analysis to explore the effects of SOAT1-targeting compounds on the cholesterol metabolism signaling pathways in tumor cells. We found that compounds targeting SOAT1 not only directly inhibited cholesterol esterification, but also inhibited cholesterol synthesis and transport and promoted cholesterol conversion (Figs. 3 and 4). Additionally, combined analysis with the omics of clinical patients demonstrated that activation or inhibition of specific cholesterol proteins are closely related to the survival of hepatocelluar carcinoma patients, and SOAT1-targeting drugs would positively facilitate the survival of patients (Fig. 5). These results systematically revealed the internal connection between cholesterol metabolic programming and tumors; specifically, SOAT1-targeting drugs reduce the synthesis, esterification, and transport of cholesterol in tumor cells. Improving cholesterol transformation in tumor cells has been shown to be beneficial to inhibiting the growth of tumor cells.

In addition, recent studies have shown that cholesterol esterase inhibitors can also enhance the antitumor effect of human chimeric antigen receptor-modified T cells [39]. These studies indicate that cholesterol metabolism also plays an important role in the activation of immune cell antitumor function. In this study, we performed multicolor flow cytometry immunoassay in an immunocompetent mouse CDX model and found that the relative quantity of CD8^**+**^ T cells and neutrophils was significantly increased in mice with tumors after two SOAT1 targeting compounds treated. Moreover, after treatment with two SOAT1-targeting compounds, tumor growth in mice was not only inhibited, but also tumor size was subsided after 2 weeks of dosing (Fig. 6). These studies confirmed that cholesterol metabolism may play an important role in the activation antitumor functions of immune cells.

## Conclusions

In summary, we screened nilotinib, a high-affinity ligand targeting the potential antitumor target SOAT1. Compared with known inhibitors, it has a higher affinity to SOAT1 and better inhibitory effect. More importantly, as a drug currently in clinical use, nilotinib can play a better antitumor effect by simultaneously targeting tumor cells and immune microenvironment by reprogramming the intracellular cholesterol metabolism of tumor cells and enhancing CD8^+^ T cells and neutrophils. It is expected to be used for targeted therapy and combined immunotherapy in patients with SOAT1^high^ hepatocellular carcinoma.

## Supplementary Information

**Additional file 1.**

**Additional file 2.**

**Additional file 3.**

**Additional file 4.**

**Additional file 5.**

**Additional file 6: Figure S1.** Schematic of the virtual screening based on SOAT1 and expression and purification of SOAT1 protein. (a) The overall workflow of the virtual screening based on the SOAT1 protein. (b, c, d) SOAT1 protein expression vector plasmid construction and restriction enzyme digestion analysis. (e) Size exclusion chromatography (SEC) purification of SOAT1 protein. (f) SDS-PAGE analysis of SOAT1 protein. The results showed that all purified SOAT1 proteins were in a stable dimer state with a purity greater than 95%. **Figure S2.** Validation of the biological activity of compounds targeting SOAT1 protein. (a) A transwell assay showed that the cell number decreased after administration with different SOAT1-targeting compounds (original magnification 100x). (b) Compounds targeting SOAT1 significantly inhibited the proliferation of liver cancer cell lines. (c) PI staining was used to investigate the effect of the ligands on the cell cycle: G0/G1 phase (blue peak), S phase (yellow peak), and G2 phase (green peak). Cells accumulated in the G0/G1 phase (n = 3). (d) Body weight of non-cell-derived xenograft models treated with sorafenib (20 mg/kg/day) or nilotinib (20 mg/kg/day) on the indicated days (n = 6 mice per group). Mean (± SD) of body weight is plotted. **Figure S3.** Proteomic sample preparation and data analysis. (a) Workflow of proteome sample preparation and data collection. (b) Scatter plots and Pearson correlation coefficients for replicate proteome profiling of two SOAT1-targeted compounds (nevanimibe and nilotinib). The x- and y-axes represent the protein intensities in each pairwise comparison. Notably, repeat experiments with the same samples have good reproducibility, with a high level of correlation (average > 0.9; range, 0.85–1). (c) Coverage of identified and quantified proteins. In total, 3,960 proteins were quantified from 7,307 identified proteins. (d) UpSet Venn diagram of each pairwise comparison. 226 differential proteins (180 downregulated and 48 upregulated) were simultaneously dysregulated in the proteome of nevanimibe and nilotinib (n=3, p<0.01). **Figure S4.** Metabolome sample preparation and data analysis. (a) Workflow of metabolome sample preparation and data collection. (b) Scatter plots and Pearson correlation coefficients for replicate metabolome profiling of two SOAT1-targeted compounds (nevanimibe and nilotinib). The x- and y-axes represent the metabolome intensities in each pairwise comparison. Notably, repeat experiments with the same samples have good reproducibility, with a high level of correlation (average > 0.9; range, 0.85–1). (c) Coverage of detected mass spectral peaks and quantitative and qualitative mass spectral. Overall, 1890 compounds were quantified from 19,671 identified MS/MS spectra, and 662 metabolites were classified. (d) UpSet Venn diagram of each pairwise comparison. 410 differential metabolites (239 downregulated and 171 upregulated) were detected in the metabolome of nevanimibe and nilotinib (n=6, p<0.01). **Figure S5.** The relationship between drug-regulated cholesterol dysregulation proteins and the survival of liver cancer patients. **Figure S6.** Schematic of multicolor flow cytometry analysis. (a) Schematic diagram of a xenograft model derived from Hepa1-6 cells transplanted into C57BL/6 mice. (b) Multicolor flow cytometry analysis of cell-derived xenograft models. (c) Histogram overlays show changes in expression profiles between samples. The table shows the groups and cell counts. (d) Fluorescence correlation analysis heat map of each color. The cells labeled with each fluorescent antibody are clearly distinguished, indicating that the analysis system is robust. **Table S1.** Four software molecar docking screenings yielded 62 potential compounds. **Table S2.** 29 cholesterol metabolism signal pathway proteins changed after administration. **Table S3.** Targeting identified 26 cholesterol metabolism signaling pathway metabolites changed after administration.

## Data Availability

The data supporting the findings of this study are available from the corresponding author upon reasonable request.

## Abbreviations

CDX: Cell-derived xenograft
DMEM: Dulbecco’s modified Eagle’s media
ESI: Electrospray ionization
FBS: Fetal bovine serum
GO: Gene Ontology
HMGCR: 3-Hydroxy-3-methylglutaryl-coenzyme A reductase
IC50: Half-maximal inhibitory concentration
KD: Equilibrium dissociation constant
KEGG: Kyoto Encyclopedia of Genes and Genomes
LC-MS: Liquid chromatography mass spectrometer
LXR: Liver X receptor
MTBE: Methyl tert-butyl ether
NPC1: NPC intracellular cholesterol transporter 1
PI: Propidium iodide
SOAT1: Sterol O-acyltransferase 1
