# Supplementary material for: High-affinity SOAT1 ligands remodeled cholesterol metabolism program to inhibit tumor growth
Source: BMC Med. 2022 Aug 9;20:292. doi: 10.1186/s12916-022-02436-8 (PMC9361549; doi:10.1186/s12916-022-02436-8)
Supplement: Supplementary file 2 — Additional file 2. [file 12916_2022_2436_MOESM2_ESM.docx]

**Original, uncropped blots of relative expression of SOAT1 in different liver cancer cell lines**

From left to right is the expression of SOAT1 protein (Figure 1) and β-actin protein (Figure 2) in Hep3B, HepG2 and Huh7 cells.

Antibodies: rabbit anti-SOAT1 polyclonal antibody (ABN66, Merck) and rabbit anti-β-actin monoclonal antibody (6609, Proteintech).

Protein Marker: Rainbow protein marker (26616, Thermo) (Figure 3)

**
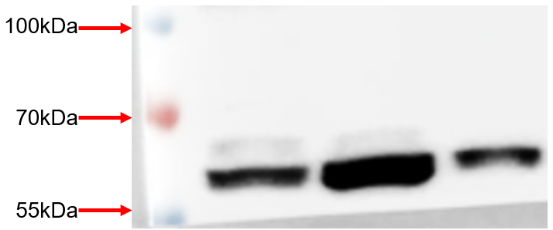
Figure 1**

**
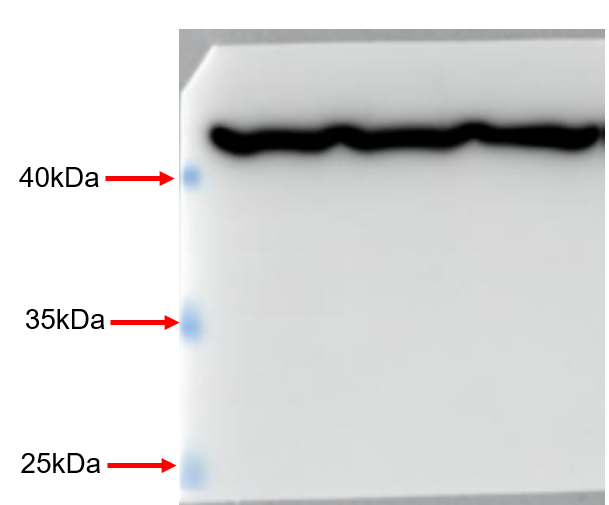
 Figure 2**


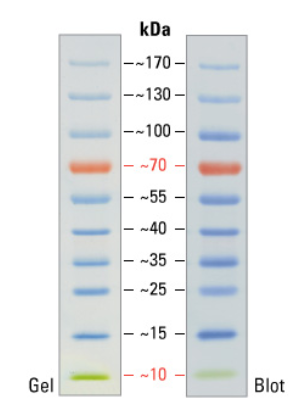
 **Figure 3**
