# Supplementary material for: High-affinity SOAT1 ligands remodeled cholesterol metabolism program to inhibit tumor growth
Source: BMC Med. 2022 Aug 9;20:292. doi: 10.1186/s12916-022-02436-8 (PMC9361549; doi:10.1186/s12916-022-02436-8)
Supplement: Supplementary file 6 — Additional file 6: Figure S1. Schematic of the virtual screening based on SOAT1 and expression and purification of SOAT1 protein. (a) The overall workflow of the virtual screening based on the SOAT1 protein. (b, c, d) SOAT1 protein expression vector plasmid construction and restriction enzyme digestion analysis. (e) Size exclusion chromatography (SEC) purification of SOAT1 protein. (f) SDS-PAGE analysis of SOAT1 protein. The results showed that all purified SOAT1 proteins were in a stable dimer state with a purity greater than 95%. Figure S2. Validation of the biological activity of compounds targeting SOAT1 protein. (a) A transwell assay showed that the cell number decreased after administration with different SOAT1-targeting compounds (original magnification 100x). (b) Compounds targeting SOAT1 significantly inhibited the proliferation of liver cancer cell lines. (c) PI staining was used to investigate the effect of the ligands on the cell cycle: G0/G1 phase (blue peak), S phase (yellow peak), and G2 phase (green peak). Cells accumulated in the G0/G1 phase (n = 3). (d) Body weight of non-cell-derived xenograft models treated with sorafenib (20 mg/kg/day) or nilotinib (20 mg/kg/day) on the indicated days (n = 6 mice per group). Mean (± SD) of body weight is plotted. Figure S3. Proteomic sample preparation and data analysis. (a) Workflow of proteome sample preparation and data collection. (b) Scatter plots and Pearson correlation coefficients for replicate proteome profiling of two SOAT1-targeted compounds (nevanimibe and nilotinib). The x- and y-axes represent the protein intensities in each pairwise comparison. Notably, repeat experiments with the same samples have good reproducibility, with a high level of correlation (average > 0.9; range, 0.85–1). (c) Coverage of identified and quantified proteins. In total, 3,960 proteins were quantified from 7,307 identified proteins. (d) UpSet Venn diagram of each pairwise comparison. 226 differential proteins (180 downregul [file 12916_2022_2436_MOESM6_ESM.docx]

**Figure S1.** **Schematic of the virtual screening based on SOAT1 and expression and purification of SOAT1 protein. (a)** The overall workflow of the virtual screening based on the SOAT1 protein. **(b, c, d)** SOAT1 protein expression vector plasmid construction and restriction enzyme digestion analysis. **(e)** Size exclusion chromatography (SEC) purification of SOAT1 protein. **(f)** SDS-PAGE analysis of SOAT1 protein. The results showed that all purified SOAT1 proteins were in a stable dimer state with a purity greater than 95%.


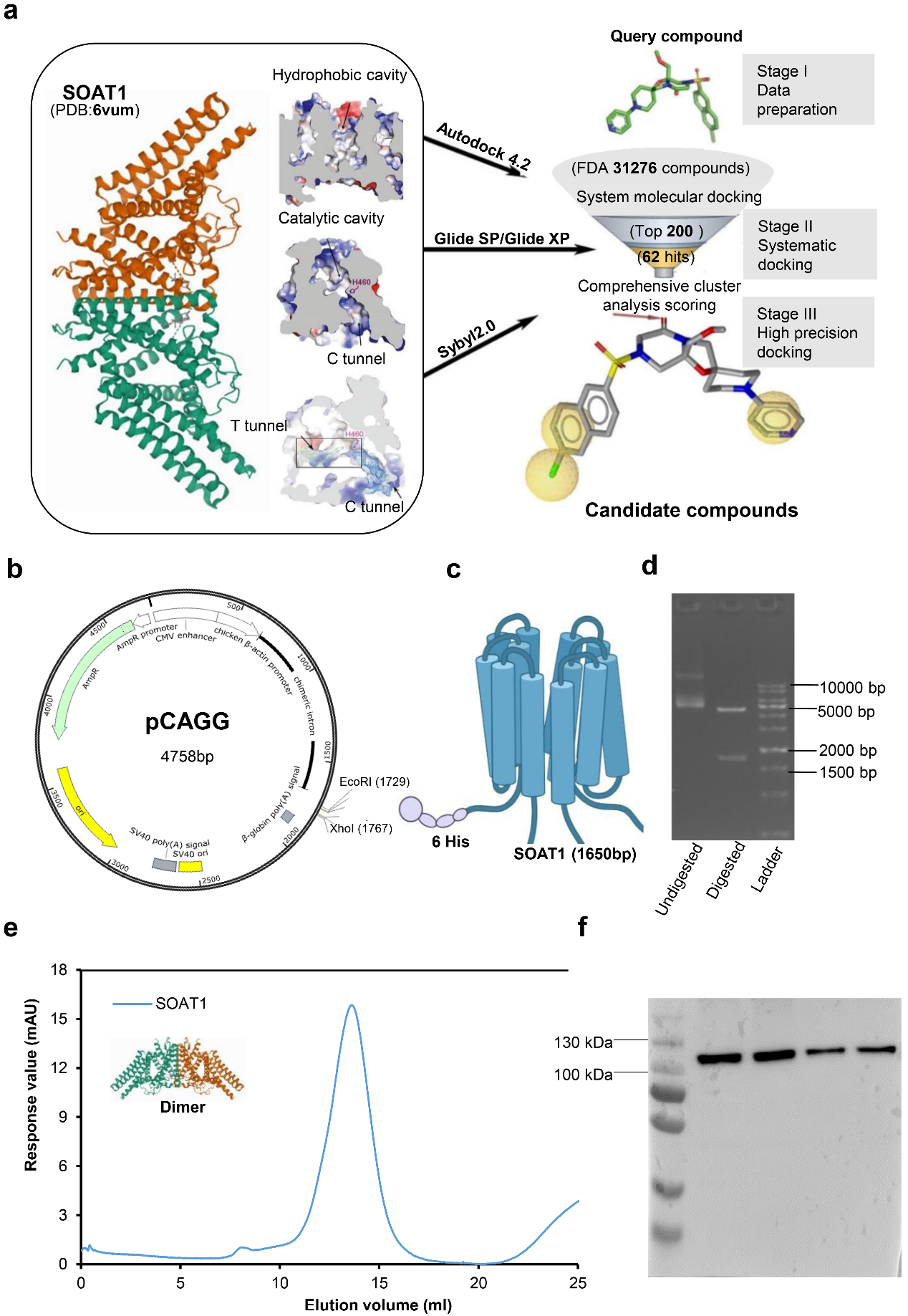


**Figure S2.** **Validation of the biological activity of compounds targeting SOAT1 protein. (a)** A transwell assay showed that the cell number decreased after administration with different SOAT1-targeting compounds (original magnification 100x). **(b)** Compounds targeting SOAT1 significantly inhibited the proliferation of liver cancer cell lines. **(c)** PI staining was used to investigate the effect of the ligands on the cell cycle: G0/G1 phase (blue peak), S phase (yellow peak), and G2 phase (green peak). Cells accumulated in the G0/G1 phase (n = 3). **(d)** Body weight of non-cell-derived xenograft models treated with sorafenib (20 mg/kg/day) or nilotinib (20 mg/kg/day) on the indicated days (n = 6 mice per group). Mean (± SD) of body weight is plotted.


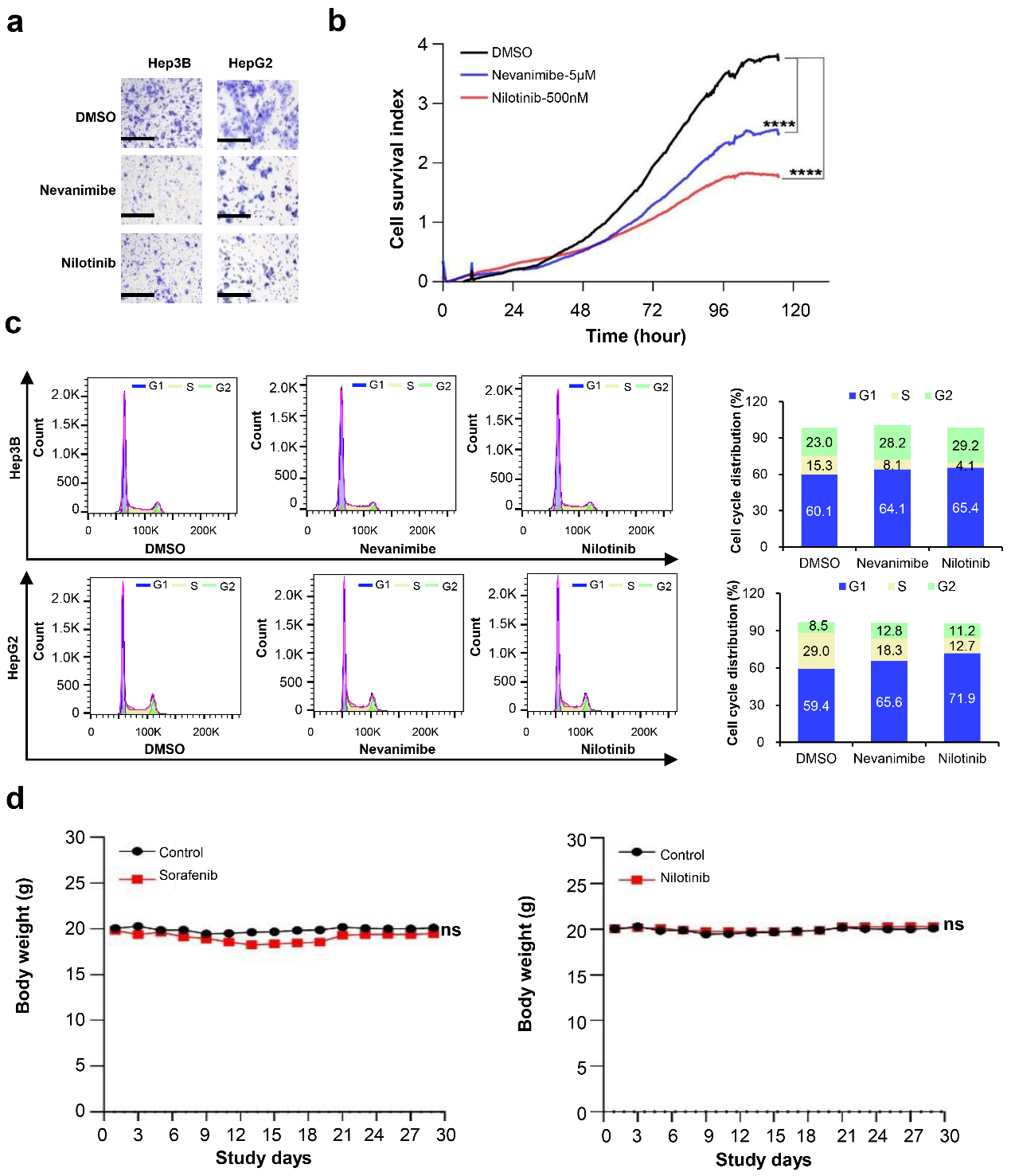


**Figure S3.** **Proteomic sample preparation and data analysis. (a)** Workflow of proteome sample preparation and data collection. **(b)** Scatter plots and Pearson correlation coefficients for replicate proteome profiling of two SOAT1-targeted compounds (nevanimibe and nilotinib). The x- and y-axes represent the protein intensities in each pairwise comparison. Notably, repeat experiments with the same samples have good reproducibility, with a high level of correlation (average > 0.9; range, 0.85–1). **(c)** Coverage of identified and quantified proteins. In total, 3,960 proteins were quantified from 7,307 identified proteins. **(d)** UpSet Venn diagram of each pairwise comparison. 226 differential proteins (180 downregulated and 48 upregulated) were simultaneously dysregulated in the proteome of nevanimibe and nilotinib (n=3, p<0.01).

**
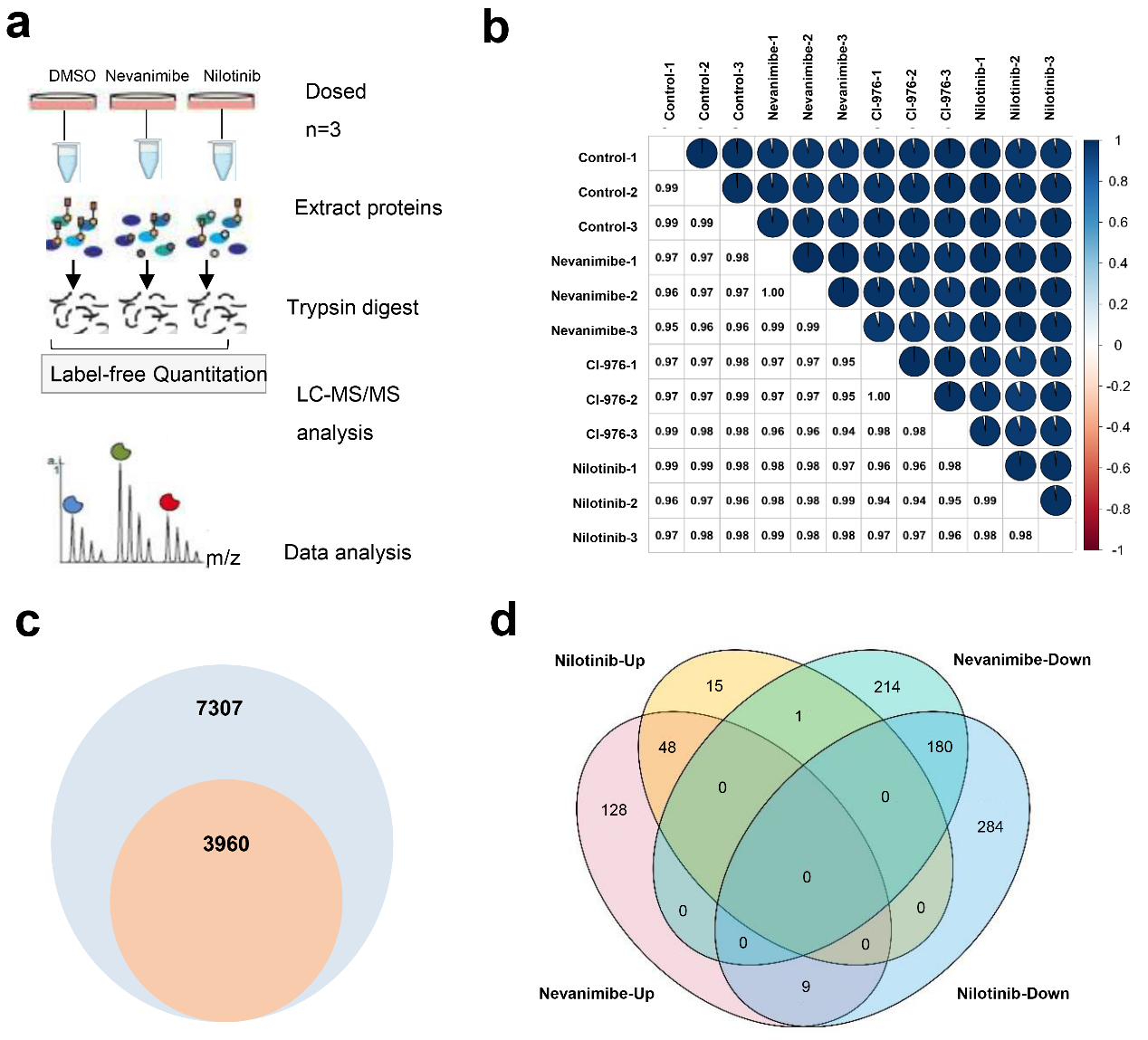
**

**Figure S4.** **Metabolome sample preparation and data analysis. (a)** Workflow of metabolome sample preparation and data collection. **(b)** Scatter plots and Pearson correlation coefficients for replicate metabolome profiling of two SOAT1-targeted compounds (nevanimibe and nilotinib). The x- and y-axes represent the metabolome intensities in each pairwise comparison. Notably, repeat experiments with the same samples have good reproducibility, with a high level of correlation (average > 0.9; range, 0.85–1). **(c)** Coverage of detected mass spectral peaks and quantitative and qualitative mass spectral. Overall, 1890 compounds were quantified from 19,671 identified MS/MS spectra, and 662 metabolites were classified. **(d)** UpSet Venn diagram of each pairwise comparison. 410 differential metabolites (239 downregulated and 171 upregulated) were detected in the metabolome of nevanimibe and nilotinib (n=6, p<0.01).

**
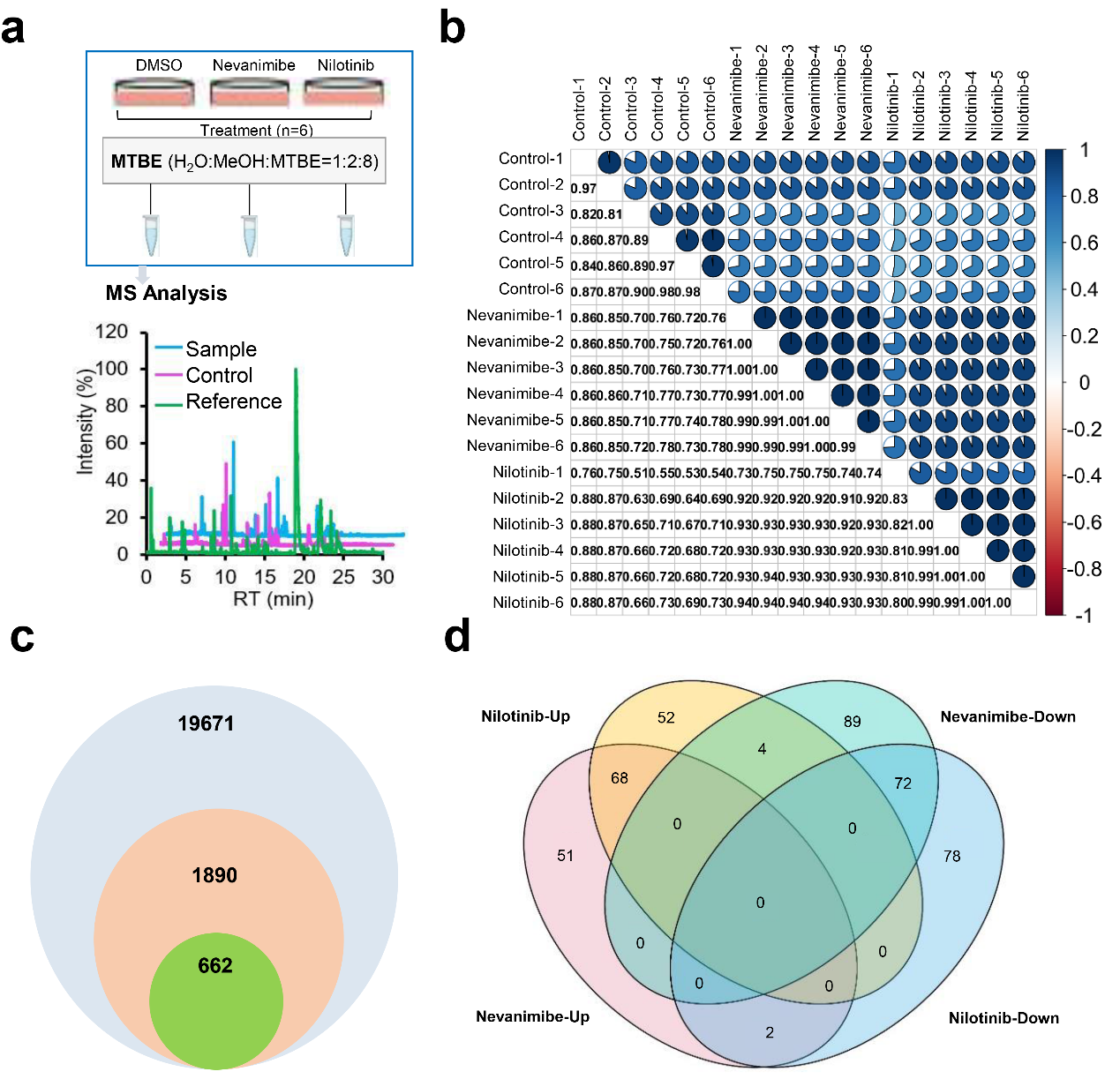
**

**Figure S5.** The relationship between drug-regulated cholesterol dysregulation proteins and the survival of liver cancer patients.

**
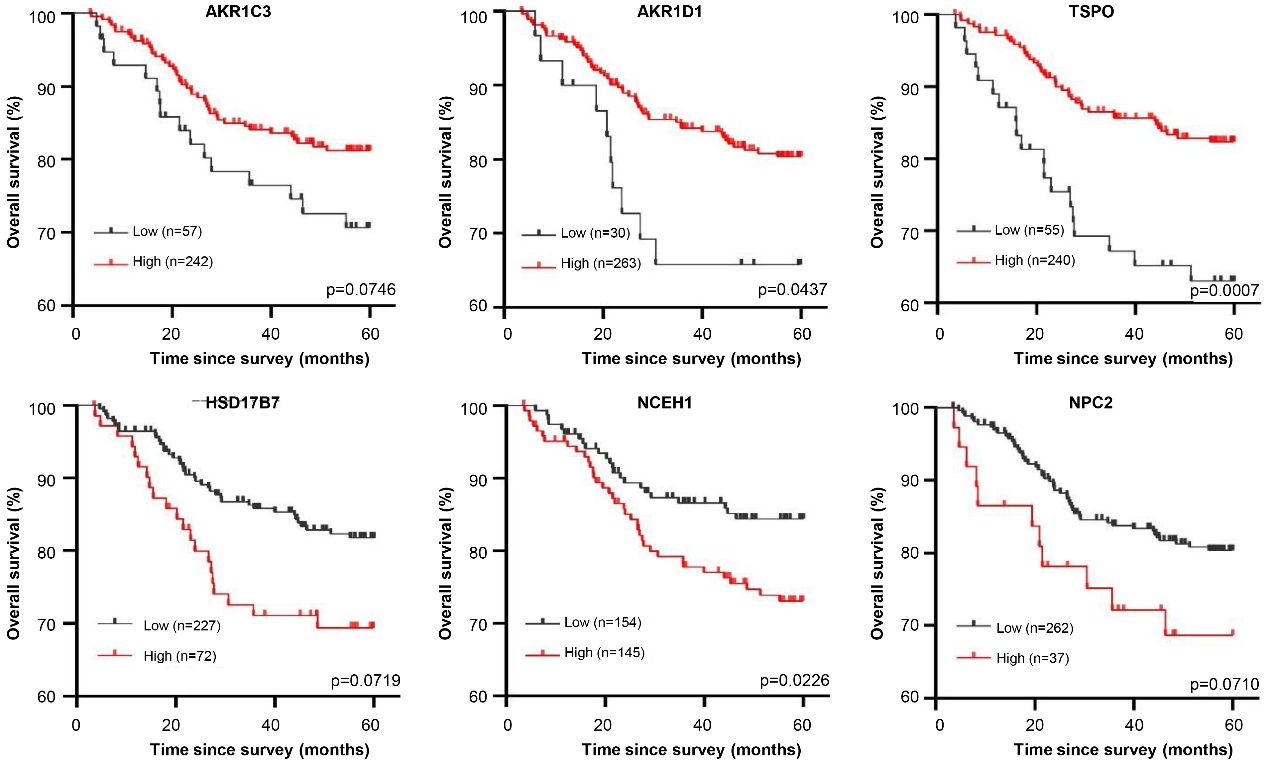
**

**Figure S6****. Schematic of multicolor flow cytometry analysis. (a)** Schematic diagram of a xenograft model derived from Hepa1-6 cells transplanted into C57BL/6 mice. (b) Multicolor flow cytometry analysis of cell-derived xenograft models. **(c)** Histogram overlays show changes in expression profiles between samples. The table shows the groups and cell counts. **(d)** Fluorescence correlation analysis heat map of each color. The cells labeled with each fluorescent antibody are clearly distinguished, indicating that the analysis system is robust.

**
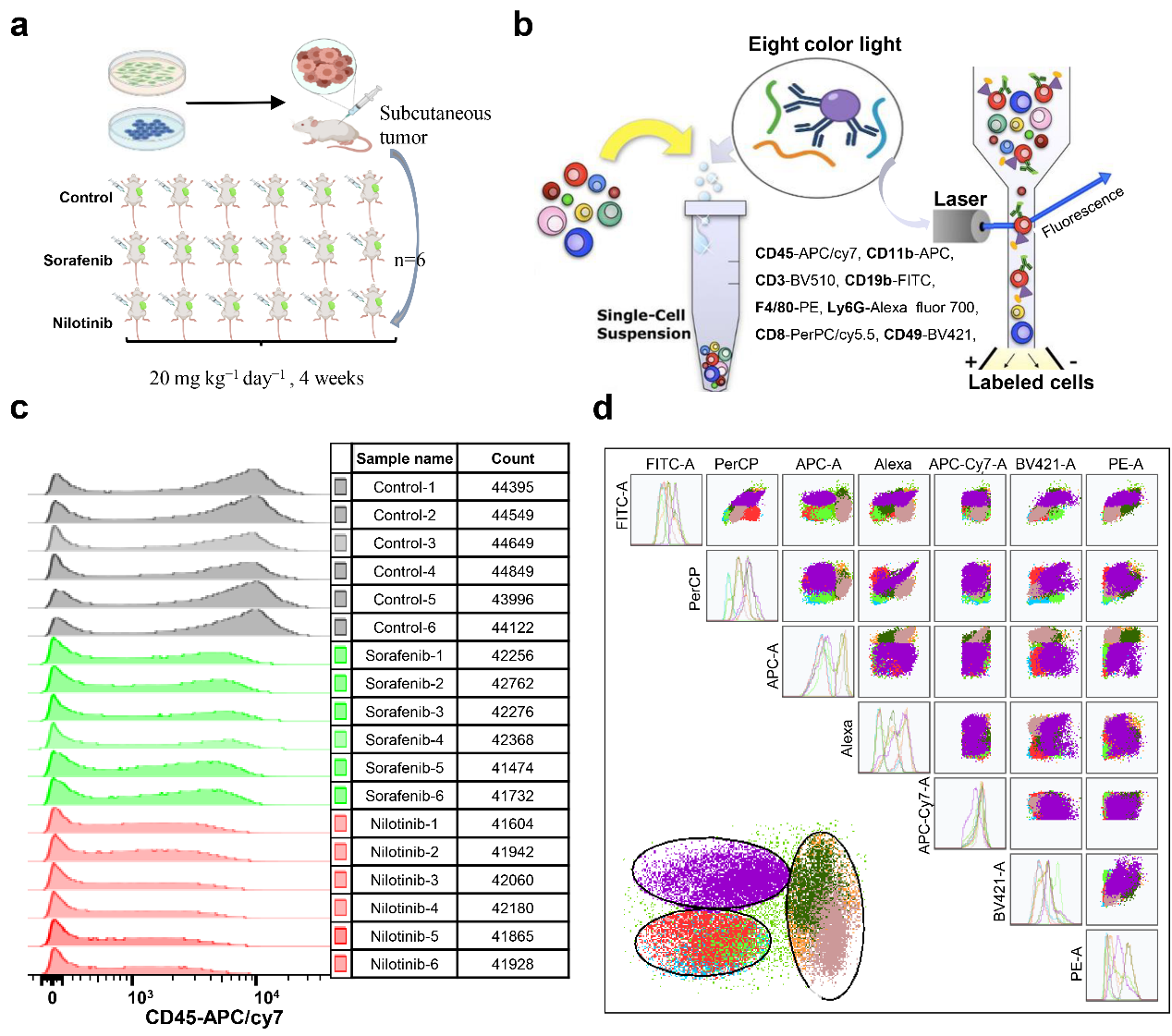
**

**Table S1**. **Four software molecar docking screenings yielded 62 potential compounds**

| **Name** | **Autodock** | **Sybyl** | **Glide SP** | **Glide XP** | **Average** | **RSD** |
| --- | --- | --- | --- | --- | --- | --- |
| SB-480848 | -10.19 | -9.91 | -9.18 | -12.07 | -10.34 | 11.92% |
| RGX-104 | -10.12 | -9.07 | -9.12 | -11.34 | -9.91 | 10.74% |
| Nilotinib | -9.69 | -8.86 | -8.87 | -10.68 | -9.52 | 9.06% |
| Verbascoside | -9.45 | -8.78 | -8.85 | -10.61 | -9.42 | 8.99% |
| Nevanimibe | -9.09 | -8.57 | -8.14 | -10.28 | -9.02 | 10.27% |
| Radotinib | -8.98 | -8.56 | -8.07 | -10.17 | -8.95 | 10.01% |
| NSC348884 | -8.89 | -8.42 | -8.01 | -10.13 | -8.86 | 10.34% |
| Indinavir Sfate | -8.88 | -8.34 | -7.97 | -10.12 | -8.83 | 10.64% |
| GNF-5837 | -8.87 | -8.34 | -7.97 | -10.05 | -8.81 | 10.33% |
| BAY-876 | -8.80 | -8.31 | -7.93 | -9.94 | -8.74 | 9.98% |
| CI-976 | -8.78 | -8.30 | -7.91 | -9.92 | -8.73 | 9.98% |
| Anacefrapib | -8.71 | -8.30 | -7.90 | -9.90 | -8.70 | 9.97% |
| Dalcetrapib | -8.55 | -8.28 | -7.87 | -9.87 | -8.65 | 10.00% |
| CB-839 | -8.51 | -8.28 | -7.82 | -9.84 | -8.61 | 10.04% |
| SPHINX31 | -8.47 | -8.22 | -7.76 | -9.75 | -8.55 | 9.95% |
| NPS-1034 | -8.45 | -8.20 | -7.72 | -9.71 | -8.52 | 9.97% |
| Ramipril | -8.43 | -8.20 | -7.67 | -9.65 | -8.49 | 9.84% |
| Montelukast | -8.42 | -8.19 | -7.66 | -9.65 | -8.48 | 9.90% |
| GW501516 | -8.39 | -8.18 | -7.66 | -9.63 | -8.47 | 9.86% |
| ME0328 | -8.38 | -8.17 | -7.65 | -9.63 | -8.46 | 9.89% |
| GW3965 HCl | -8.28 | -8.12 | -7.65 | -9.56 | -8.40 | 9.73% |
| ABT-737 | -8.27 | -8.08 | -7.62 | -9.54 | -8.38 | 9.82% |
| BMS-927711 | -8.24 | -8.07 | -7.60 | -9.53 | -8.36 | 9.87% |
| URB597 | -8.24 | -8.06 | -7.57 | -9.52 | -8.35 | 9.99% |
| ICG-001 | -8.21 | -8.04 | -7.56 | -9.48 | -8.32 | 9.85% |
| PRI-724 | -8.21 | -8.04 | -7.55 | -9.44 | -8.31 | 9.65% |
| GLPG0634 | -8.18 | -8.04 | -7.53 | -9.42 | -8.29 | 9.70% |
| SB-715992 | -8.14 | -7.98 | -7.42 | -9.39 | -8.23 | 10.07% |
| SRT2183 | -8.09 | -7.92 | -7.34 | -9.32 | -8.17 | 10.22% |
| LXR-625 | -8.08 | -7.90 | -7.33 | -9.32 | -8.16 | 10.23% |
| Z-DEVD-FMK | -8.07 | -7.89 | -7.33 | -9.30 | -8.15 | 10.19% |
| Evacetrapib | -7.94 | -7.83 | -7.27 | -9.19 | -8.06 | 10.07% |
| BT-11 | -7.94 | -7.81 | -7.27 | -9.18 | -8.05 | 10.04% |
| NVP-BHG712 | -7.92 | -7.80 | -7.26 | -9.15 | -8.03 | 9.92% |
| Terbinafine | -7.92 | -7.79 | -7.25 | -9.15 | -8.03 | 9.95% |
| Ebastine | -7.87 | -7.79 | -7.22 | -9.13 | -8.00 | 10.08% |
| Fostemsavir | -7.84 | -7.75 | -7.20 | -9.10 | -7.97 | 10.06% |
| ODM-201 | -7.84 | -7.73 | -7.19 | -9.09 | -7.96 | 10.11% |
| TGR-1202 | -7.84 | -7.71 | -7.18 | -9.05 | -7.94 | 9.96% |
| PD123319 | -7.82 | -7.70 | -7.16 | -9.03 | -7.93 | 9.95% |
| R788 | -7.81 | -7.68 | -7.15 | -9.01 | -7.91 | 9.95% |
| Simvastatin | -7.80 | -7.68 | -7.13 | -9.00 | -7.90 | 9.98% |
| MYCi975 | -7.79 | -7.67 | -7.12 | -8.99 | -7.89 | 9.98% |
| TAK-285 | -7.79 | -7.66 | -7.12 | -8.98 | -7.89 | 9.91% |
| Pexmetinib | -7.79 | -7.66 | -7.11 | -8.96 | -7.88 | 9.87% |
| 666-15 | -7.77 | -7.64 | -7.10 | -8.94 | -7.86 | 9.85% |
| NP-G2-044 | -7.66 | -7.51 | -6.98 | -8.77 | -7.73 | 9.76% |
| LXH254 | -7.65 | -7.50 | -6.97 | -8.75 | -7.72 | 9.67% |
| Betin | -7.59 | -7.47 | -6.94 | -8.69 | -7.67 | 9.57% |
| NAD+ | -7.57 | -7.46 | -6.92 | -8.68 | -7.66 | 9.67% |
| Lapatinib | -7.56 | -7.45 | -6.91 | -8.66 | -7.65 | 9.59% |
| MYCi361 | -7.52 | -7.42 | -6.88 | -8.62 | -7.61 | 9.60% |
| SR9243 | -7.49 | -7.36 | -6.85 | -8.57 | -7.57 | 9.53% |
| GSK621 | -7.48 | -7.36 | -6.85 | -8.56 | -7.56 | 9.51% |
| Ro-48-8071 | -7.48 | -7.36 | -6.85 | -8.56 | -7.56 | 9.50% |
| GS-444217 | -7.47 | -7.35 | -6.83 | -8.52 | -7.54 | 9.42% |
| NUC-1031 | -7.46 | -7.34 | -6.81 | -8.50 | -7.53 | 9.38% |
| KN-62 | -7.43 | -7.32 | -6.79 | -8.47 | -7.50 | 9.39% |
| AZ628 | -7.42 | -7.32 | -6.78 | -8.46 | -7.50 | 9.37% |
| Tucidinostat | -7.42 | -7.31 | -6.78 | -8.46 | -7.49 | 9.40% |
| Anacetrapib | -7.41 | -7.29 | -6.77 | -8.45 | -7.48 | 9.39% |
| Avasimibe | -7.34 | -7.26 | -6.71 | -8.41 | -7.43 | 9.54% |

*****Choose the top 200 compounds from the screening results of each software and be screened out by at least three software, all the results with Avasimibe as the control.

**Table S2**. **29 cholesterol metabolism signal pathway proteins changed after administration.**

| Accession | Gene name | p-Value | Log2(FC)-nenanimibe | Log2(FC)-nilotinib |
| --- | --- | --- | --- | --- |
| O95477 | ABCA1 | 1.44E-04 | -2.01 | -2.52 |
| P42330 | AKR1C3 | 2.52E-05 | 2.93 | 2.48 |
| P51857 | AKR1D1 | 1.07E-01 | 2.27 | 1.99 |
| P02649 | APOE | 2.68E-03 | -1.98 | -1.77 |
| P05108 | CYP11A1 | 2.65E-04 | 1.81 | 2.11 |
| P05093 | CYP17A1 | 3.18E-05 | 2.13 | 1.89 |
| Q02318 | CYP27A1 | 6.95E-07 | 1.41 | 2.20 |
| Q16850 | CYP51A1 | 1.18E-06 | -1.41 | -1.39 |
| Q9UBM7 | DHCR7 | 3.14E-02 | -3.77 | -2.23 |
| P37268 | FDFT1 | 9.32E-06 | -1.78 | -1.43 |
| Q53GQ0 | HSD17B12 | 2.40E-05 | -1.33 | -1.81 |
| Q9H2F3 | HSD3B7 | 4.21E-03 | 1.76 | 1.64 |
| P13473 | LAMP2 | 2.33E-03 | -1.26 | -1.84 |
| P01130 | LDLR | 2.19E-03 | -2.74 | -2.05 |
| Q9BU23 | LMF2 | 4.64E-04 | -2.87 | -1.87 |
| Q07954 | LRP1 | 1.84E-03 | -1.57 | -1.79 |
| P48449 | LSS | 5.79E-06 | -2.70 | -2.41 |
| P55157 | MTTP | 1.36E-03 | -2.73 | -2.33 |
| Q6PIU2 | NCEH1 | 8.79E-05 | -3.03 | -2.64 |
| O15118 | NPC1 | 7.31E-04 | -2.38 | -1.90 |
| P61916 | NPC2 | 1.32E-03 | -1.73 | -1.82 |
| Q9H4L5 | OSBPL3 | 6.00E-02 | 2.53 | 1.34 |
| P55058 | PLTP | 1.45E-02 | -1.34 | -1.12 |
| Q15126 | PMVK | 1.57E-05 | -2.62 | -2.48 |
| P35610 | SOAT1 | 3.47E-04 | -2.31 | -2.05 |
| Q14534 | SQLE | 1.99E-03 | -2.46 | -1.97 |
| O76062 | TM7SF2 | 3.25E-03 | -3.77 | -2.59 |
| P30536 | TSPO | 4.31E-04 | 1.39 | 2.01 |
| P98155 | VLDLR | 4.87E-03 | -1.73 | -2.12 |

*The value represents Log_2_ (FC), n=3, p<0.01, FC=Compound/DMSO.

**Table S3. Targeting identified 26 cholesterol metabolism signaling pathway metabolites changed after administration.**

| **Metabolite name** | **Formula** | **Mz** | **Rt(min)** | **Nevanimibe** | **Nilotinib** |
| --- | --- | --- | --- | --- | --- |
| Squalene | C30H50 | 411.3985 | 3.70 | -6.24 | -5.54 |
| Lanosterol | C30H50O | 427.3820 | 3.08 | -8.72 | -7.39 |
| 7-Dehydrocholesterol | C27H44O | 385.3120 | 13.34 | -1.27 | -1.30 |
| 14-Demethyllanosterol | C29H48O | 413.3778 | 0.75 | -2.75 | -2.24 |
| 4,4-Dimethyl-5α-cholesta-8-en-3β-ol | C29H50O | 415.3934 | 0.85 | -1.98 | -1.19 |
| Cholesterol | C27H46O | 387.3621 | 2.20 | -2.27 | -2.61 |
| Estrone | C18H22O2 | 271.1723 | 1.33 | 3.61 | 2.29 |
| Pregnenolone | C21H32O2 | 317.2262 | 0.81 | 1.93 | 3.09 |
| Lithocholic Acid | C24H40O3 | 377.3048 | 2.28 | 1.69 | 2.59 |
| chenodeoxycholic acid | C24H40O4 | 785.6241 | 8.05 | 3.47 | 4.53 |
| Deoxycholic acid | C24H40O4 | 393.3177 | 0.94 | 0.16 | 1.18 |
| hyodeoxycholic acid | C24H40O4 | 785.5850 | 13.51 | 3.42 | 4.06 |
| glycohyodeoxycholic acid | C26H43NO5 | 921.6090 | 7.13 | 2.16 | 1.97 |
| taurocholic acid | C26H45NO7S | 498.2869 | 1.79 | 4.83 | 1.81 |
| Vitamin D2 | C28H44O | 397.3262 | 3.03 | 2.20 | 2.37 |
| 24-hydroxycholesterol | C27H46O2 | 403.3571 | 0.71 | 1.57 | 1.26 |
| 25-hydroxycholesterol | C27H46O2 | 403.3571 | 1.58 | 1.11 | 2.11 |
| 27-Hydroxycholesterol | C27H46O2 | 403.3363 | 2.09 | 3.07 | 1.58 |
| 20α,22β-Dihydroxycholesterol | C27H46O3 | 419.0298 | 28.01 | 2.17 | 2.47 |
| 4,4-Dimethyl-5α-cholesta-8,14,24-trien-3β-ol | C29H46O | 411.3621 | 0.71 | 1.30 | 1.40 |
| CE 16:0 | C43H76O2 | 647.5694 | 5.28 | -1.74 | -2.87 |
| CE 18:0 | C45H80O2 | 675.6094 | 7.03 | -10.87 | -10.39 |
| CE 20:0 | C47H84O2 | 703.6315 | 8.21 | -3.21 | -4.44 |
| CE 22:0 | C49H88O2 | 731.6706 | 12.71 | -12.47 | -10.25 |
| CE 24:0 | C51H92O2 | 759.6982 | 13.65 | -12.32 | -7.03 |
| CE 25:0 | C52H94O2 | 773.7280 | 14.01 | -9.63 | -3.37 |

*The value represents Log_2_ (FC), n=6, p<0.01, RSD<30%, FC=Compound/DMSO.
